# Genome-Wide Reprogramming of Transcript Architecture by Temperature Specifies the Developmental States of the Human Pathogen *Histoplasma*

**DOI:** 10.1371/journal.pgen.1005395

**Published:** 2015-07-15

**Authors:** Sarah A. Gilmore, Mark Voorhies, Dana Gebhart, Anita Sil

**Affiliations:** Department of Microbiology and Immunology, Howard Hughes Medical Institute, University of California, San Francisco, San Francisco, California, United States of America; Duke University Medical Center, UNITED STATES

## Abstract

Eukaryotic cells integrate layers of gene regulation to coordinate complex cellular processes; however, mechanisms of post-transcriptional gene regulation remain poorly studied. The human fungal pathogen *Histoplasma capsulatum* (*Hc*) responds to environmental or host temperature by initiating unique transcriptional programs to specify multicellular (hyphae) or unicellular (yeast) developmental states that function in infectivity or pathogenesis, respectively. Here we used recent advances in next-generation sequencing to uncover a novel re-programming of transcript length between *Hc* developmental cell types. We found that ~2% percent of *Hc* transcripts exhibit 5’ leader sequences that differ markedly in length between morphogenetic states. Ribosome density and mRNA abundance measurements of differential leader transcripts revealed nuanced transcriptional and translational regulation. One such class of regulated longer leader transcripts exhibited tight transcriptional and translational repression. Further examination of these dually repressed genes revealed that some control *Hc* morphology and that their strict regulation is necessary for the pathogen to make appropriate developmental decisions in response to temperature.

## Author Summary

Eukaryotic cells alter their developmental programs in response to environmental signals. *Histoplasma capsulatum* (*Hc*), a ubiquitous fungal pathogen of humans, establishes unique transcriptional programs to specify growth in either a multicellular hyphal form or unicellular yeast form in response to temperature. Since hyphae and yeast are specialized to function in infectivity or pathogenesis, respectively, *Hc* provides a clinically relevant system in which to query eukaryotic regulatory processes. Here we used next-generation sequencing approaches to annotate the transcriptomes of four distinct *Hc* strains in response to temperature. We found that a fraction of *Hc* transcripts have differential transcript architecture in hyphae and yeast, exhibiting 5’ leader sequences that differ markedly in length between morphogenetic states. To begin to understand the effect of these differential leader sequences on expression, we performed the first ribosome density and mRNA abundance measurements in *Hc*, thereby uncovering transcriptional and translational control that contribute to cell-type regulation.

## Introduction

**Fig 1:**
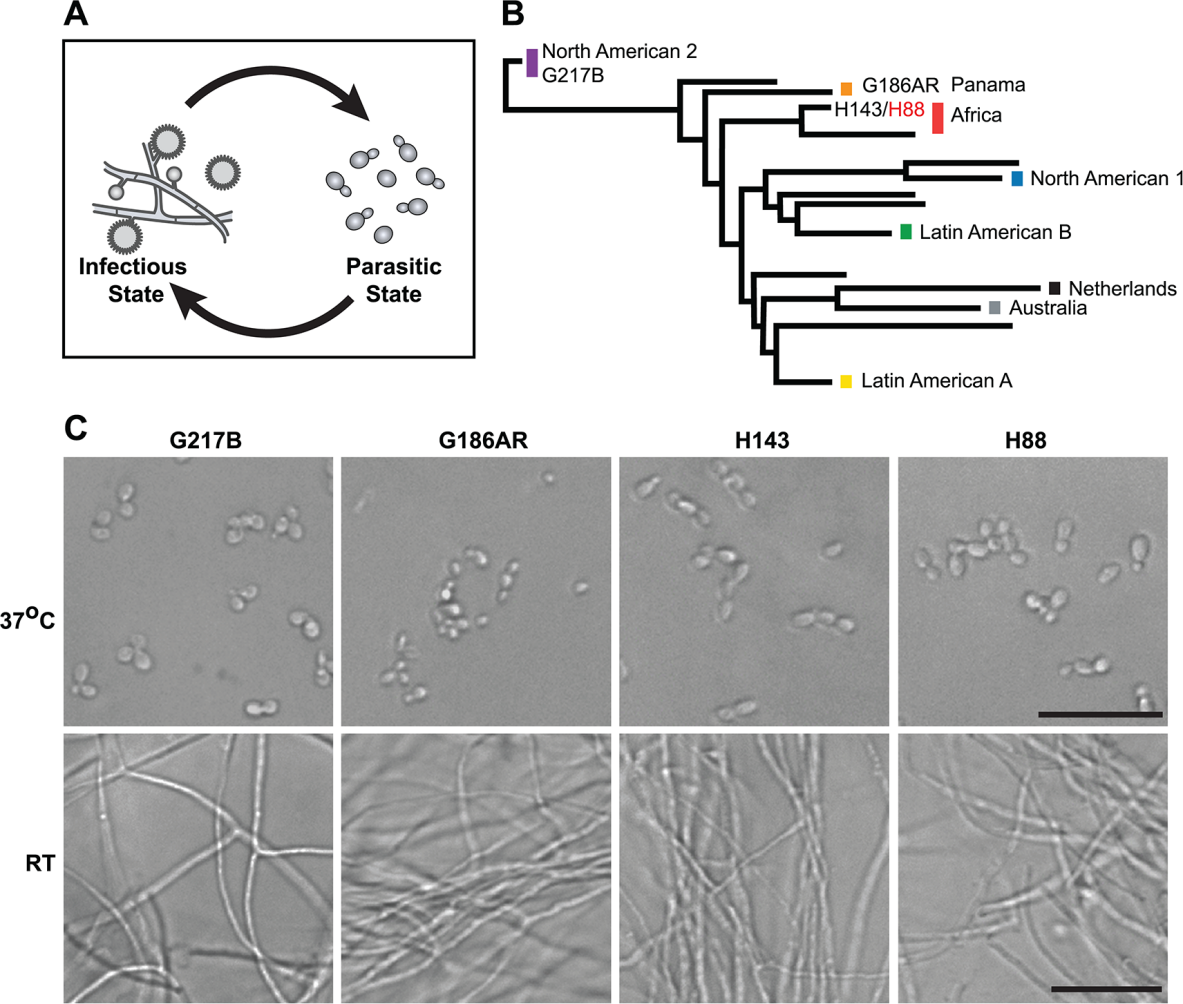
*Histoplasma* is a global human pathogen with distinct morphological states important for virulence. (A) *Histoplasma* hyphae and conidia (infectious state) respond to temperature to differentiate into budding yeast cells (parasitic state) inside of a mammalian host. (B) Phylogeny of *Histoplasma* illustrating the major lineages of this species, which are geographically and genetically distinct. This neighbor-joining tree was adapted from Katsuga *et al*. [7] and highlights *Histoplasma* strains selected for comparative transcriptomics (G217B, G186AR, H88, H143). H88 is highlighted in red as the *Hc* var. *duboisii* strain selected for transcriptome analysis. (C) Confocal DIC microscopy showing representative images of yeast (37°C) and hyphal (RT) cell morphologies for the 4 *Hc* strains used for comparative transcriptomics analyses. Scale bar, 20 μm.

Environmental human pathogens have evolved the ability to survive in human hosts as well as diverse environmental reservoirs. Thus a hallmark of environmental pathogens is their capacity to adapt to varied growth conditions such as differences in temperature, alterations in nutrient sources, as well as exposure to the host immune system. The dimorphic human fungal pathogen, *Histoplasma capsulatum* (*Hc*), is one such environmental pathogen that responds to an increase in temperature inside a mammalian host by dramatically altering its cellular morphology and gene expression programs to cause disease. The environmental, infectious form of *Hc* grows in the soil as a saprophyte in a multicellular hyphal or “mold” form that produces vegetative spores called conidia [1,2]. Infection occurs when humans inhale conidia or hyphal fragments. Upon exposure to mammalian body temperature, *Hc* transitions into a unicellular, budding yeast (**Fig 1A**) that is capable of causing primary disease in immunocompetent individuals [3–6]. The temperature-regulated differentiation of *Hc* between the hyphal and yeast forms can be recapitulated in the laboratory simply by switching the temperature from room temperature (RT) to 37°C, making *Hc* a unique organism for studying the regulation of gene expression during multicellular development, environmental signal transduction, and adaptation of a pathogen to a mammalian host.

*Hc* cells respond to temperature through a transcriptional regulatory circuit that controls cell morphology as well as the expression of 5–15% of the *Hc* transcriptome that is differentially regulated between yeast and hyphal cells [8–10]. In addition, transcripts encoding many of the known *Hc* virulence factors are enriched in the yeast-phase of growth [11], making yeast-enriched transcripts intriguing virulence factor candidates. To this end, microarrays [8,9,12–14] and RNA-sequencing (RNA-seq) [10] have begun to elucidate the transcriptomes of yeast, hyphal, and conidial cell types to describe transcript expression patterns that specify these three major cell types important for the *Hc* pathogenic lifecycle. Despite these efforts, our understanding of the *Hc* transcriptome and cell-morphology specific regulatory programs remains incomplete partly due to challenges inherent in deciphering intron-rich, densely populated eukaryotic fungal transcriptomes.

To date, *Hc* transcriptomics studies have been performed using two North and Central American *Hc* var. *capsulatum* strains, G217B and G186AR (see **Fig 1B**). Yet, *Hc* is found globally (North, South, and Central America, Southeast Asia, and Africa) and phylogenetic analysis of *Hc* clinical isolates has revealed that *Hc* comprises at least 8 genetically and geographically distinct lineages that span 3–13 million years of evolutionary distance [7,15] (**Fig 1B**). In addition to its genetic diversity, *Hc* can be segregated into two varieties (var.) based on clinical outcomes: *Hc* var. *duboisii* and *Hc* var. *capsulatum*. *Hc* var. *capsulatum* is represented in all of the major *Hc* lineages and infection with this variety predominantly manifests as pulmonary and systemic disease [4]. *Hc* var. *duboisii*, conversely, is geographically restricted to Western and Central Africa and causes cutaneous and subcutaneous skin as well as bone lesions in a systemic infection that is distinguished in name from classical disease as African histoplasmosis [16,17]. The virulence mechanisms and basic biology of the causative agent of African histoplasmosis, *Hc* var. *duboisii*, as well as *Hc* var. *capsulatum* strains from additional, more divergent lineages, remain uninvestigated.

In this work, we employ recent advances in next-generation sequencing and *de novo* transcriptome reconstruction methodologies to refine transcript architecture and define transcript expression programs across divergent isolates of *Hc* that cause classical (G217B, G186AR, H143) or African (H88) histoplasmosis. Using comparative transcriptomics of two temperature-regulated states, we broaden our understanding of the basic biology and pathogenesis of *Hc* by defining core yeast (parasitic, disease-causing form) and hyphal (infectious form) transcript expression patterns that exhibit conserved regulatory patterns. Systematic analysis of our improved *Hc* transcript architecture further revealed that a subset of the *Hc* transcriptome (~ 2%) exhibited dramatically different leader sequence lengths (often referred to as the 5’ untranslated region; UTR) between these two *Hc* developmental cell states. To begin to understand the biological significance of longer leader transcripts, we probed their ribosome occupancy using ribosome profiling. Consequently, we defined several categories of genes, including those that are regulated at both the transcriptional and translational level in response to temperature-initiated developmental programs. Phenotypic analysis revealed that this exquisite regulation is necessary for appropriate cell-type specification by temperature, the key signal for infectious and parasitic states.

## Results

### Comparative annotation of *Histoplasma* transcriptomes

**Fig 2:**
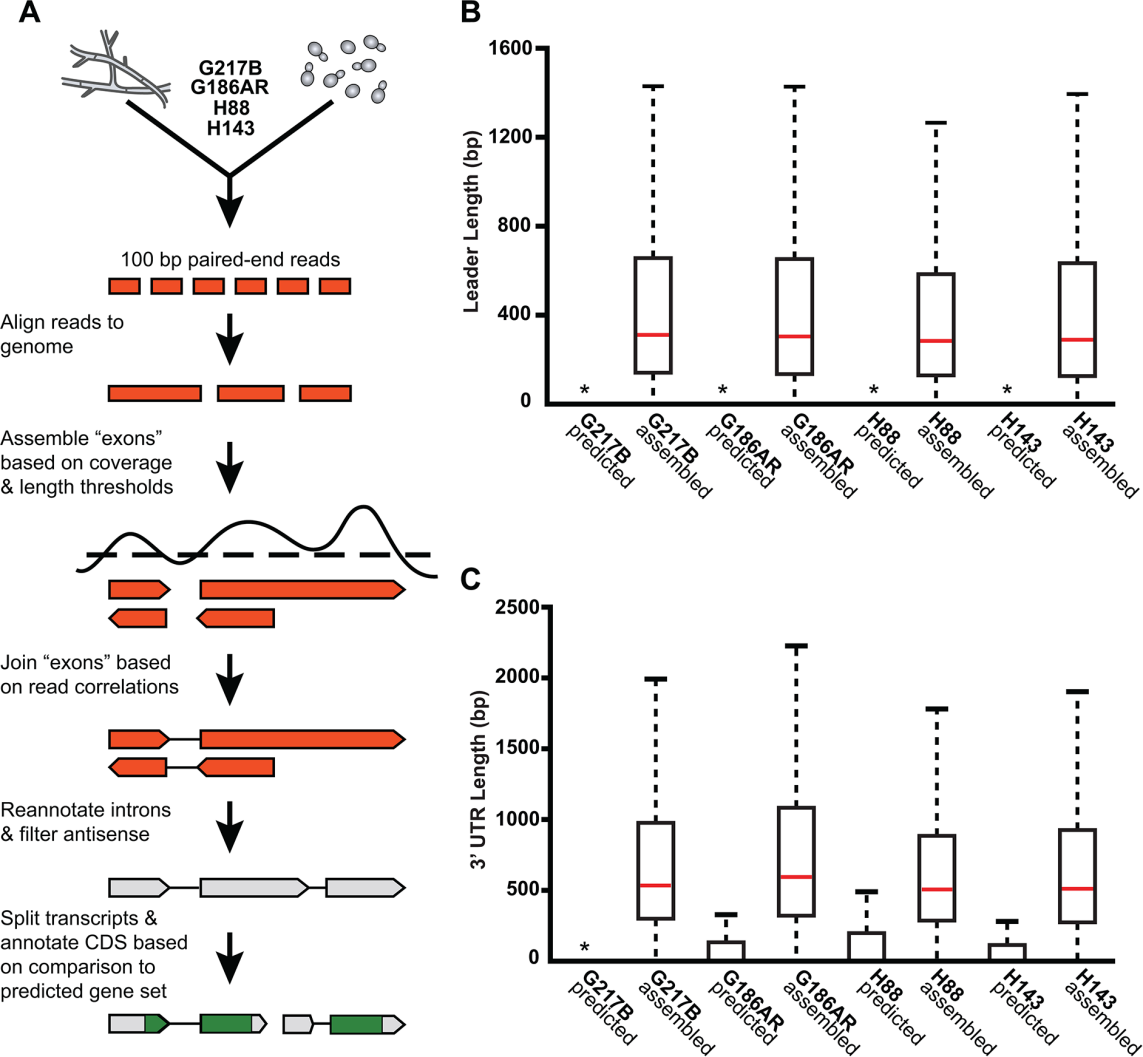
*De novo Histoplasma* transcriptome reconstruction augments transcript models. (A) Schematic of transcriptome reconstruction method. (B–C) Boxplots of the length of (B) leader regions that were defined as the distance from the 5’ transcript end to the CDS start codon or (C) 3’ UTR regions that were defined as the distance from the CDS stop codon to the 3’ transcript end were plotted for all assembled and predicted transcripts with CDS regions > 0. Boxes, interquartile range (IQR). Whiskers, 1.5*IQR. For ease of viewing, outliers are not displayed. *, indicates that the predicted transcript leader or 3’ UTR length distributions is not visible on this graph. The predicted transcript leader lengths range from 0–1652 bp and the 3’ UTR lengths range from 0–3112 bp in the 4 *Hc* strains.

To compare yeast and hyphal transcript expression patterns across *Hc* lineages, we grew each *Hc* strain (G217B, G186AR, H88, H143) at 37°C for yeast-phase growth and at room temperature (RT) for hyphal phase growth (**Fig 1C**). Total RNA was isolated from biological duplicates of yeast or hyphal cultures and strand specific cDNA libraries were created from poly(A)-enriched RNA and sequenced using paired-end deep-sequencing. The 4 *Hc* strains that we selected for transcriptomics analysis each have sequenced genomes; however, the *Hc* gene predictions vary among strains and often lack accurate models of 5’ leader and 3’ UTR regions. Therefore we constructed *de novo* transcript calls leveraging our paired-end RNA-seq data with the aim of improving transcript models, generating high confidence ortholog mapping across *Hc* strains, and directly identifying poly(A)+ transcribed regions. To do so we combined yeast and hyphal sequencing reads for each *Hc* strain and used these pooled reads to generate per-strain reference transcriptomes across the 4 *Hc* lineages (see **Fig 2A**; **S1 Table**; **S1–S12 Data**; Materials and Methods). *Hc* RNA species without polyadenylated 3’ ends (*i*.*e*., potential small non-coding RNA) were not enriched in our sequencing libraries and thus are likely not represented in these transcript models.

Our genome-guided transcriptome assembly pipeline yielded 12, 175–12, 889 transcripts for the 4 *Hc* strains with 9, 580–9, 844 of assembled transcripts predicted to be protein coding (**Table 1**). This falls within the range of 9, 229–11, 329 protein coding genes predicted from *Hc* genomic sequences by *ab initio* gene prediction algorithms (**Table 1**). These *Hc* transcript models do not take into account any alternate transcript isoforms that may exist within or between *Hc* cell types (*i*.*e*., transcript forms that arise due to intron retention); however this is an interesting area for future refinement and investigation. The assembled transcripts were overall larger in size than the predicted transcript set (**S1 Fig**), which is mostly due to the improved annotation of 5’ leader and 3’ UTR transcript ends (**Fig 2B and 2C**). In addition to augmenting 5’ and 3’ transcript end annotations, we noticed many cases where assembled transcripts had more accurate intron boundaries than predicted transcripts resulting in improved annotation of coding sequence (CDS) regions (**S2 Fig**).

**Table 1:** Comparison of numbers and orthology mapping methods of assembled and predicted transcripts.

	G217B	G186AR	H88	H143
Predicted Transcripts	11, 330	9, 233	9, 428	9, 532
Predicted Protein Coding Transcripts	11, 329	9, 229	9, 424	9, 483
Predicted InParanoid Ortholog Pairs (G217B)		7, 104	7, 122	6, 831
Predicted Mercator Orthogroups (per strain)	7, 485	7, 960	8, 610	8, 325
Assembled Transcripts	12, 313	12, 663	12, 175	12, 889
Assembled Protein Coding Transcripts	9, 580	9, 844	9, 647	9, 723
Assembled InParanoid Ortholog Pairs (G217B)		6, 708	6, 670	6, 079
Assembled Mercator Orthogroups (per strain)	7, 362	7, 659	8, 288	7, 819
1:1:1:1 Mercator Orthogroups	6, 791	6, 791	6, 791	6, 791

In order to compare transcription across strains, groups of orthologous genes among the 4 *Hc* strains (orthogroups) were determined using two independent methods, Mercator [18] and InParanoid [19], which gave similar numbers of per-strain ortholog assignments (**Table 1**). For downstream analyses, we chose the Mercator method for determining *Hc* orthogroups since it incorporates both BLASTP [20] homology and genomic synteny for a more stringent determination of orthogroups. Using Mercator we assembled 6, 791 unique *Hc* orthogroups that were present in all four strains (see Materials and Methods; **Table 1**). Genes not assigned to orthogroups by this method could include in-paralogs (gene duplications that occurred after strain divergence), improperly assembled transcripts that led to inappropriate open reading frame (ORF) predictions, and genes truly unique to a given strain. Of the 6, 791 *Hc* orthogroups, 423 (6.2%) were not in any of the previously predicted *Hc* transcript sets and exhibited a striking pattern of short CDS regions relative to overall transcript lengths (**S3 Fig**). Only a small percentage of these novel orthogroups had conserved Pfam-A domains (4%) [21] or predicted secretion signals (9% as determined by Phobius [22,23]), and they exhibited lower transcript expression levels in comparison to the full orthogroup set (**S4 Fig**). In other eukaryotic species, poly(A)+ long non-coding RNAs (lncRNAs) often have lower expression levels than protein-encoding transcripts [24]. Thus, this set of novel orthogroups in *Hc* may include cases of poly(A)+ non-coding RNA species with spurious ORF predictions as well as small proteins or peptides missed by *ab initio* genome prediction algorithms. Overall, however, 90% of *Hc* orthogroups were predicted to encode at least one Pfam-A domain [21] indicating that the vast majority of *Hc* orthogroups determined from our RNA-seq transcriptome assemblies represent bona fide protein coding transcripts.

### Identification of evolutionarily conserved programs of gene regulation across *Histoplasma* infectious and parasitic cell types

We used eXpress [25] to determine transcript expression levels across yeast and hyphal cell morphologies for the 4 *Hc* strains. eXpress estimates of FPKM values (fragments per kilobase of exon per million aligned fragments) were well-correlated between biological replicates (**S2 Table**); therefore we took the mean of log_2_ FPKM values of biological replicates for downstream analyses of *Hc* yeast and hyphal expression patterns (**S13 Data**). To define a global picture of evolutionarily conserved programs of gene regulation across *Hc* lineages, we identified transcripts enriched in the yeast (parasitic form) or hyphal (infectious form) state from the 6, 791 *Hc* orthogroups (**S5 Fig**; **S13–S15 Data**). Minor lineage-specific differences in gene expression were observed, though these were largely of unknown biological significance. In terms of conserved patterns of gene expression, we found 139 yeast-phase enriched transcripts (core yeast-phase transcripts: Y/H log_2_ ≥ 1.5) and 291 hyphal-phase enriched transcripts (core hyphal-phase transcripts: Y/H log_2_ ≤ - 1.5) conserved in differential expression pattern among the 4 *Hc* strains (**S5B and S5C Fig**; **S13 Data**). Core yeast-phase enriched transcripts included two characterized virulence factors (*CBP1*, *SOD3*) [26,27], siderophore biosynthesis and transport genes (*SID3*, *SID4*, *ABC1*) important for yeast cell iron acquisition [28], and 6 putative transcription factors (*CSR1*, *CHA4*, *MEA1*, *RYP1*, *RYP4*, *XBP1*) including *RYP1* and *RYP4*, which are master transcriptional regulators of *Hc* yeast-phase growth [8,9]. We also identified 8 cell wall modifying enzymes (*AMY2*, *CFP8*, *CTS1*, *DCW1*, *ENG1*, *GEL2*, *OCH1*, *SKN1*) with transcripts upregulated in the yeast phase that are predicted to be involved in the biosynthesis or remodeling of cell wall polysaccharides such as β-glucan, chitin, and mannan; the upregulation of these transcripts in yeast may reflect differences inherent to yeast versus hyphal cell division and growth. Core hyphal-phase enriched transcripts included 18 putative transcription factors (see **S13 Data**), many of which are uncharacterized and are members of the fungal-specific Zn_2_C_6_ DNA binding domain family of transcriptional regulators (Pfam Accession: PF00172). In addition, we noticed that many core hyphal-phase enriched transcripts encoded enzymes such as cytochrome p450s, polyphenol oxidases (also known as tyrosinases), oxidoreductases, and peroxidases (see **S13 Data**). The hyphal-phase enrichment of these enzymes, which are often involved in nutrient acquisition or the production of toxins, melanin, and other secondary metabolites, is likely reflective of the saprophytic lifestyle of *Hc* hyphal cells.

### Analysis of the parasitic-phase transcriptome reveals expansion of knottins, putative effectors of *Hc* virulence

**Fig 3:**
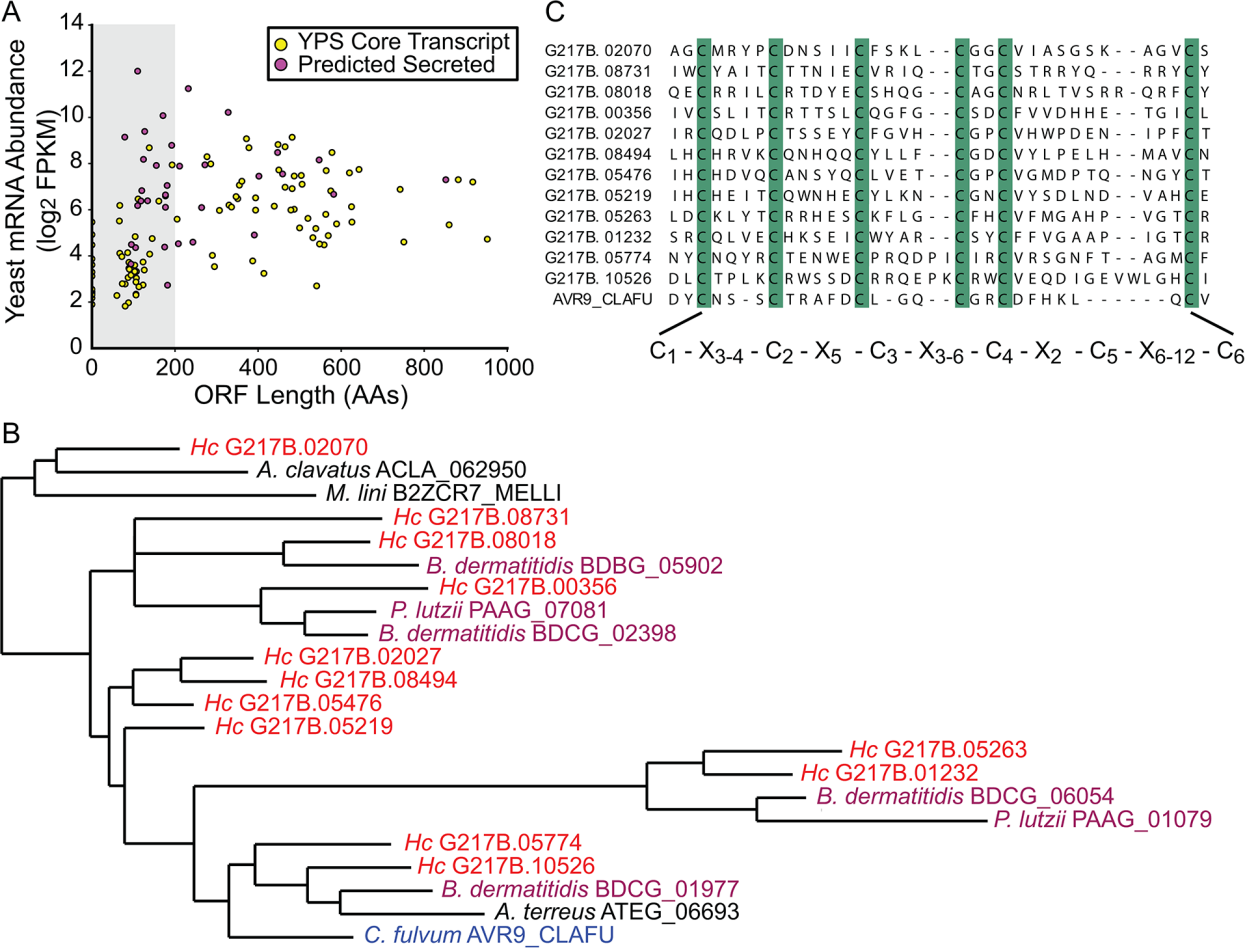
The *Histoplasma* transcriptome encodes many small, cysteine-rich proteins with homology to a knottin gene family. (A) G217B yeast mRNA abundance (log_2_ FPKM) is plotted against ORF length (number of amino acids, AA) for the core, conserved yeast-enriched transcript set. Transcripts that encode ORFs ≤ 200 AAs are located in a grey-shaded rectangle. Transcripts that encode ORFs predicted to be secreted are highlighted in magenta, whereas the remainder of the YPS core transcripts are shaded yellow. Outlier ORFs > 1000 AAs (n = 3) are not shown on this plot for ease of viewing. Similar plots for all 4 *Hc* strains are shown in S6 Fig. (B) Some *Hc* proteins with a conserved 6-cysteine spacing pattern show homology to a knottin gene family. An abbreviated phylogenetic tree of fungal knottin proteins is shown. Species in the Onygenales order of Ascomycetes are highlighted in red (*Hc* strains) or purple (all other Onygenales species) and the previously characterized *C*. *fulvum* knottin protein, Avr9, is highlighted in blue. *A*. *clavatus*; *Aspergillus clavatus*. *M*. *lini*; *Melampsora lini*. *B*. *dermatitidis*; *Blastomyces dermatitidis*. *P*. *lutzii*; *Paracoccidioides lutzii*. *A*. *terreus*; *Aspergillus terreus*. *C*. *fulvum*; *Cladosporium fulvum*. The complete aligned tree with local tree node support values is given in S7 Fig. (C) Alignment of the C-terminus of G217B knottin proteins with the characterized Avr9 knottin protein from *Cladosporium fulvum* [33]. Cysteine residues are highlighted in green and a consensus cysteine spacing pattern derived from these sequences is shown below the alignment. *Hc* transcript names are truncated from ucsf_hc.01_1.X, where X = transcript name shown.

Examining the relative abundances of yeast-enriched transcripts (using per-state mRNA FPKMs) indicated that some of the most abundant core yeast-enriched transcripts (*CBP1*, *GH17*/*CFP4*, *SOD3*, *ENG1*) encode proteins known to be secreted by yeast cells [29] (**S6 Fig**). Since bacterial and fungal pathogens often utilize secreted proteins as virulence effectors [30,31], we were interested in identifying additional conserved yeast-phase enriched transcripts encoding proteins with secretion signals. Prediction of secretion signal peptides for the conserved yeast-phase enriched ORFs (using Phobius [22,23]) revealed that secreted proteins were significantly more likely to be differentially expressed than non-secreted proteins (p = 0.000314), suggesting that many secreted proteins could have phase-specific roles. In addition, we noticed that there were many small (≤ 200 AAs) putative secreted proteins (**Figs 3A** and **S6**) that exhibited a conserved C-terminal, 6-cysteine spacing pattern reminiscent of some insect toxins [32].

We examined existing hidden Markov models (HMM) of cysteine-rich protein domains to determine whether this *Hc* 6-cysteine motif belonged to any known protein families. Through this analysis, we found that many *Hc* ORFs with the 6-cysteine motif had homology to a cystine knot (or knottin) gene family [34]. Knottin domains are comprised of 3 interwoven disulfide bonds that form one of the smallest known stable globular domains [35], making these proteins extremely resistant to chemical, heat, and proteolytic stresses [36]. Knottins can be found in fungi, insects, plants, and animals [34], and their 3-disulfide bond core can present constrained loop structures that mediate protein-protein interactions [37]. Functionally, knottin proteins have been shown to act as pore formers, ion-channel inhibitors, as well as protease inhibitors [38–40]. Phylogenetic analysis of proteins with identifiable knottin domains (of the Fungi1 knottin family [34,41]) in *Hc* as well as other related fungal species, revealed that this family appears to have undergone an expansion in the Ajellomycetaceae family of human fungal pathogens (*Hc*, *Paracoccidioides brasiliensis*, *Blastomyces dermatitidis)* (**Figs 3B** and **S7**; **S16–S18 Data**). Notably, knottins are absent from many Saccharomycetes fungi such as *Saccharomyces cerevisiae and Candida albicans* (**S7 Fig**). *Hc* transcripts encoding this knottin domain are predominantly yeast-phase enriched (**S8 Fig**) and are intriguing candidates for virulence effectors that could mediate host-pathogen interactions.

### Transcriptome assembly across *Histoplasma* lineages uncovers genome-wide regulation of transcript length between cell morphologies

**Fig 4:**
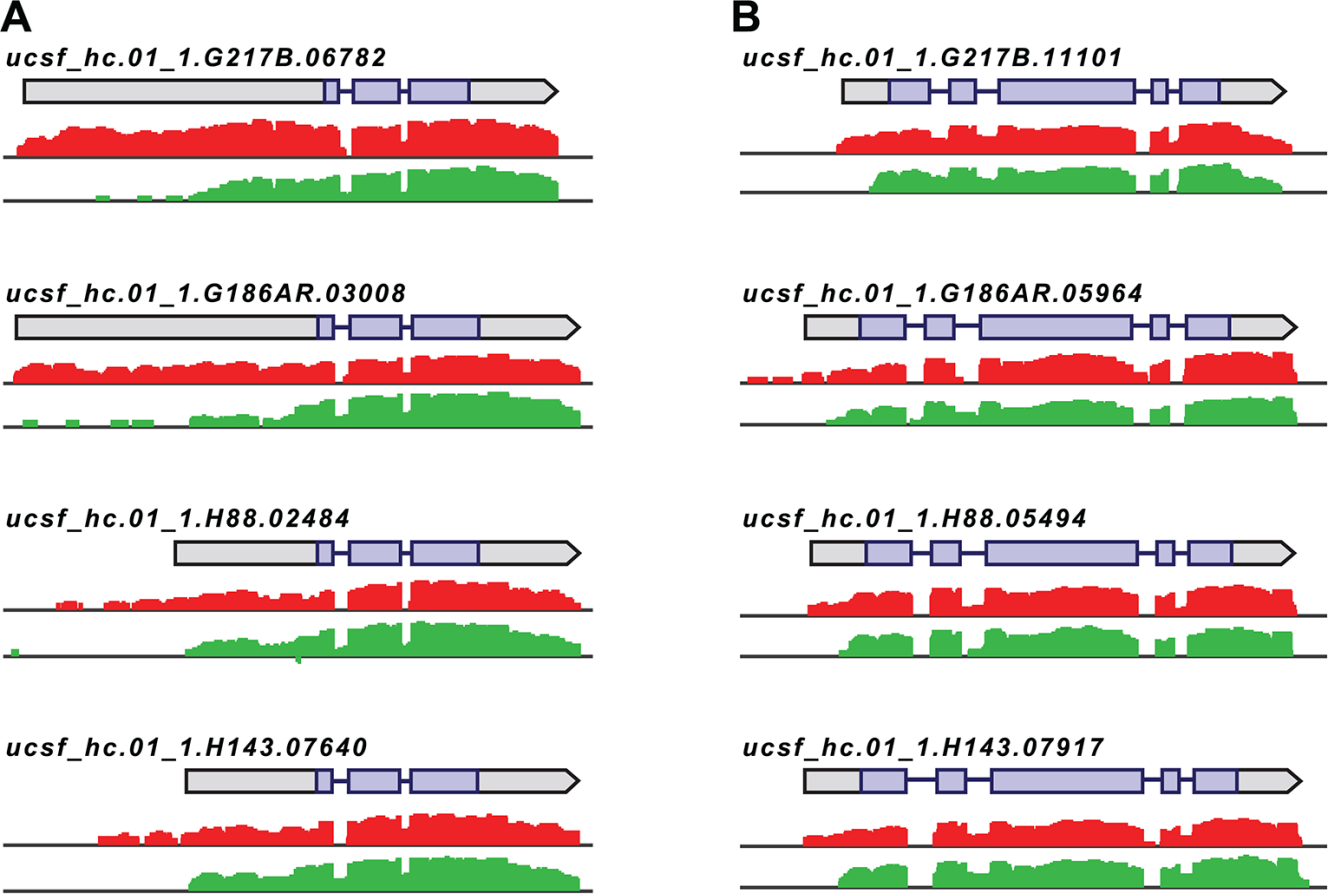
*Histoplasma* regulates 5’ transcript length in yeast and hyphal cells. Paired-end mRNA read coverage is shown for two representative transcripts (A and B) that exhibit extended yeast-phase leader regions in all 4 *Hc* strains. Log_2_ yeast read coverage is plotted in red and log_2_ hyphal read coverage is plotted in green. The assembled transcript structures are indicated above the read coverage tracks with the predicted CDS region highlighted in purple. For simplicity, only the genomic region and strand encompassing each transcript are displayed; this figure does not show an alignment at the genomic nucleotide level between all 4 *Hc* strains.

While examining our improved annotation of transcript boundaries in the 4 assembled *Hc* transcriptomes, we noticed that a subset of transcripts exhibited differences in the size of their leader regions between yeast and hyphal cell types. For example, we observed that some transcripts in yeast cells displayed longer leader regions as compared to their cognate hyphal transcripts (**Fig 4**). This was a phenomenon that we had previously seen for a handful of *Hc* yeast-enriched transcripts while examining transcript abundance and architecture in G217B yeast and hyphal cells using Northern blotting and 5’ RACE (5’ rapid amplification of cDNA ends) [13]. Given the high-resolution nature of RNA-seq data, we were able to identify leader regions that were differential in length between *Hc* morphologies genome-wide across all 4 strains. To do so, we employed our existing transcript assembly pipeline to determine per-cell type (yeast and hyphae) transcript structures for each *Hc* strain (see Materials and Methods; **S19–S26 Data**). Globally, we found that most leader regions were similar in length between yeast and hyphae in all 4 *Hc* strains as expected (**S9**and **S10 Figs**). However, systematic analysis of leader regions using per-cell type assembled transcript structures identified 187 transcripts with conserved, differentially sized leader regions between yeast and hyphal cells (see Materials and Methods; **S10 Fig**; **S13 Data**). The conservation of differential leader transcript structure in all 4 *Hc* lineages examined suggests that changes to 5’ transcript length are not a stochastic transcriptional outcome, but are evolutionarily conserved and likely regulated by the cell.

We found that the majority of transcripts that exhibited differential leader length are longer in the yeast phase compared to the hyphal phase of *Hc* growth (138/187 differential leaders; **S10 Fig**; **S13 Data)**. We did, however, find 49/187 conserved transcripts with longer leaders in hyphal cells (**S10 Fig**; **S13 Data**), indicating that differential leader length is not an artifact of sample preparation or produced exclusively by one *Hc* cell type. We examined the sizes of a subset of longer leader transcripts in yeast and hyphal cells via Northern blotting, which confirmed the differential transcripts sizes between *Hc* cell morphologies (**S11 Fig**). Northern blot analysis of longer leader transcripts also highlighted that some of these transcripts represent a population of transcripts of variable sizes (**S11 Fig**). This is analogous to emerging observations of complex transcript structure in *S*. *cerevisiae* where variations in 5’ and 3’ transcript boundaries give rise to many different transcript isoforms for a given gene [42,43].

### Establishment of ribosome profiling in *Histoplasma* yeast and hyphae to probe translational regulation of longer leader transcripts

**Fig 5:**
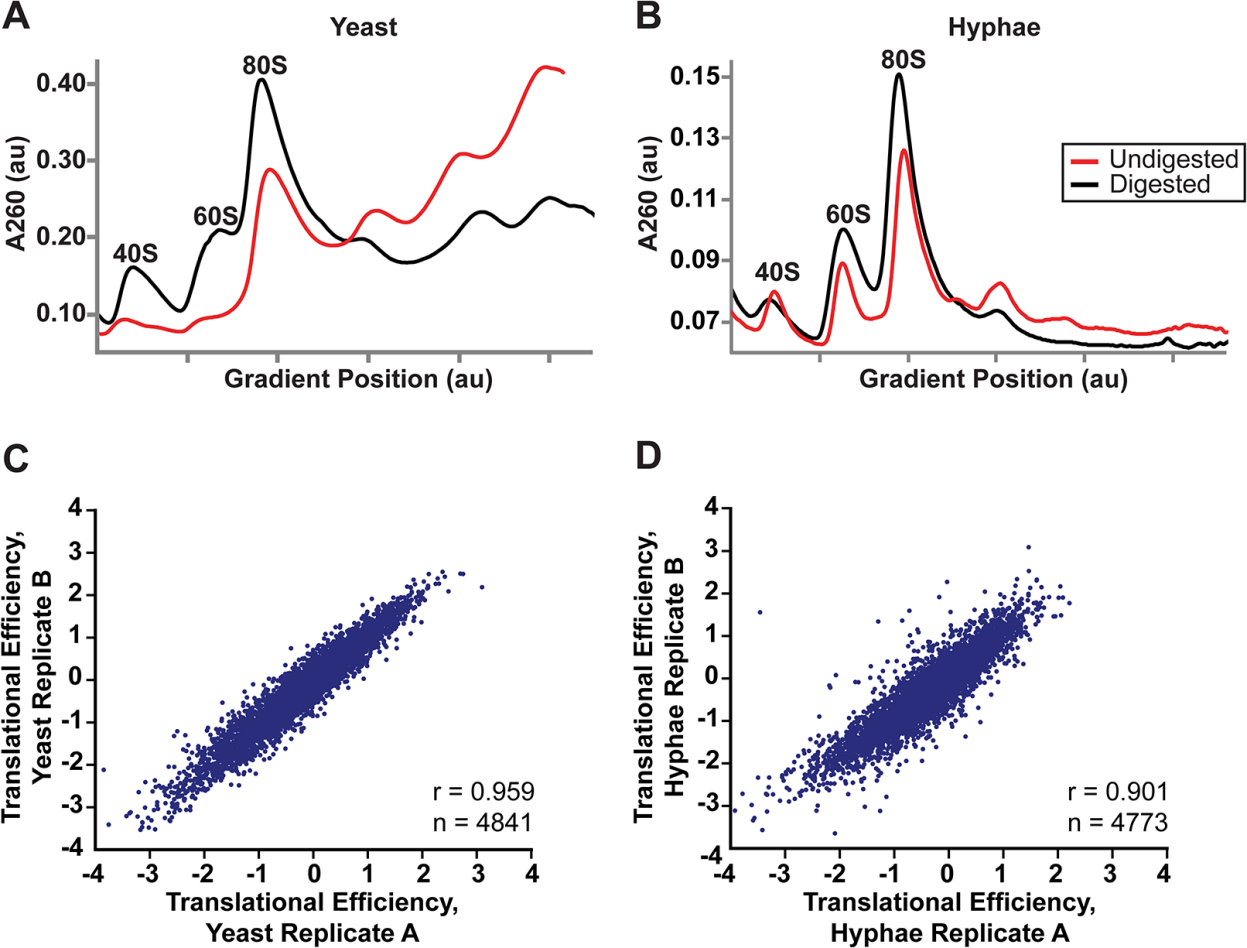
Successful adaptation of ribosome profiling to *Histoplasma*. (A–B) 80S monosome peaks were collected from RNaseI-digested yeast (A) and hyphal (B) cell extracts by fractionation over a 10–50% sucrose gradient (black line). Undigested polysome profiles (red line) are shown overlaid on the same graph which plots UV absorbance at 260 nm versus gradient position during fractionation of the sucrose gradient. au, arbitrary units. (C–D) Pearson correlation coefficients (adjusted r values) are given for linear regressions of log_2_ translational efficiency measurements of biological replicates of yeast (C) or hyphae (D). Only genes with summed footprint counts in coding regions ≥ 128 between replicates were used in these comparisons. One-way ANOVA p values were below the machine rounding threshold (< 2.2 x 10^−16^).

Differential sizes of transcript leader regions between cell states or in cells encountering changing environmental conditions is a recently observed genome-wide phenomenon in eukaryotes [44–48]. While the biological function of differential leader regions is often unclear, changes in leader length have been proposed to influence the translational outcome of protein coding ORFs either by introducing regions of RNA that can serve to regulate translation (*e*.*g*., upstream ORFs; uORFS) [44] or by introducing an alternate start codon that encodes a new protein variant [42]. Thus we hypothesized that differential leader transcripts in *Hc* may be translationally regulated between *Hc* developmental states. To investigate this possibility we adapted ribosome profiling to *Hc*. Ribosome profiling is based on deep-sequencing of ribosome-protected RNA fragments or “footprints” and has been well-established as a method to probe translational regulation in eukaryotic cells [44,49–51]. Analogous to ribosome profiling developed in the model yeast organism *S*. *cerevisiae* [49], we digested total RNA from yeast or hyphal G217B *Hc* cells with RNase I and then separated and fractionated the RNA with a sucrose density gradient to collect *Hc* 80S monosomes (**Fig 5A and 5B**). Polysome profiles of undigested RNA from yeast and hyphal vegetative cells were noticeably different (**Fig 5A and 5B**), most likely illustrating the distinct modes of growth for these *Hc* cell types. *Hc* yeast grow and divide as unicellular budding yeast cells while *Hc* hyphal cells are connected by septa to form filamentous, branched hyphae that grow as highly polarized multicellular structures. In Ascomycete fungi, hyphal cells exhibit specialization and often possess differences in growth rate, cell size, or access to nutrients [52], leading to a heterogeneous mix of cell states per hyphae.

We collected biological replicate samples of yeast and hyphae monosomes as well as poly(A)-enriched RNA from the same samples for ribosome profiling and RNA-seq, respectively. Ribosome footprint density and mRNA abundance measurements (calculated as the number of ribosome-protected or mRNA fragments per kilobase of coding sequence per million mapped reads; FPKM) were reproducible between yeast and hyphal biological replicates (**S12 Fig**). With ribosome profiling data we can also determine the translational efficiency (TE) of transcripts (calculated as the ratio of ribosome footprint density to mRNA abundance over a coding region), which is a measure of the extent to which a transcript is translationally regulated. Importantly, measurements of TE in *Hc* were reproducible between biological replicates (**Fig 5C and 5D**; **S27 Data**) and TE measurements for genes expressed in yeast or hyphal cells spanned similar orders of magnitude (**S13A Fig**). Additionally, ribosome footprint FPKMs for transcripts involved in basic cellular functions thought to be important for growth or maintenance of both cells types (*ACT1*, *GAPDH*, *TEF1*, *CDC2*, *TUB1*, and *TUB2*) were overall remarkably similar between yeast and hyphae (**S13B Fig**; **S27 Data**). However, we want to stress that the ribosome profiling data sets provide relative measures of translational efficiencies and not absolute measures of translation. Together these data indicate that ribosome profiling is a robust method to probe genome-wide ribosome occupancy as well as the translational regulation of transcripts in the developmentally distinct morphologies of *Hc*.

### Translational control contributes to specification of the yeast and hyphal developmental programs

**Fig 6:**
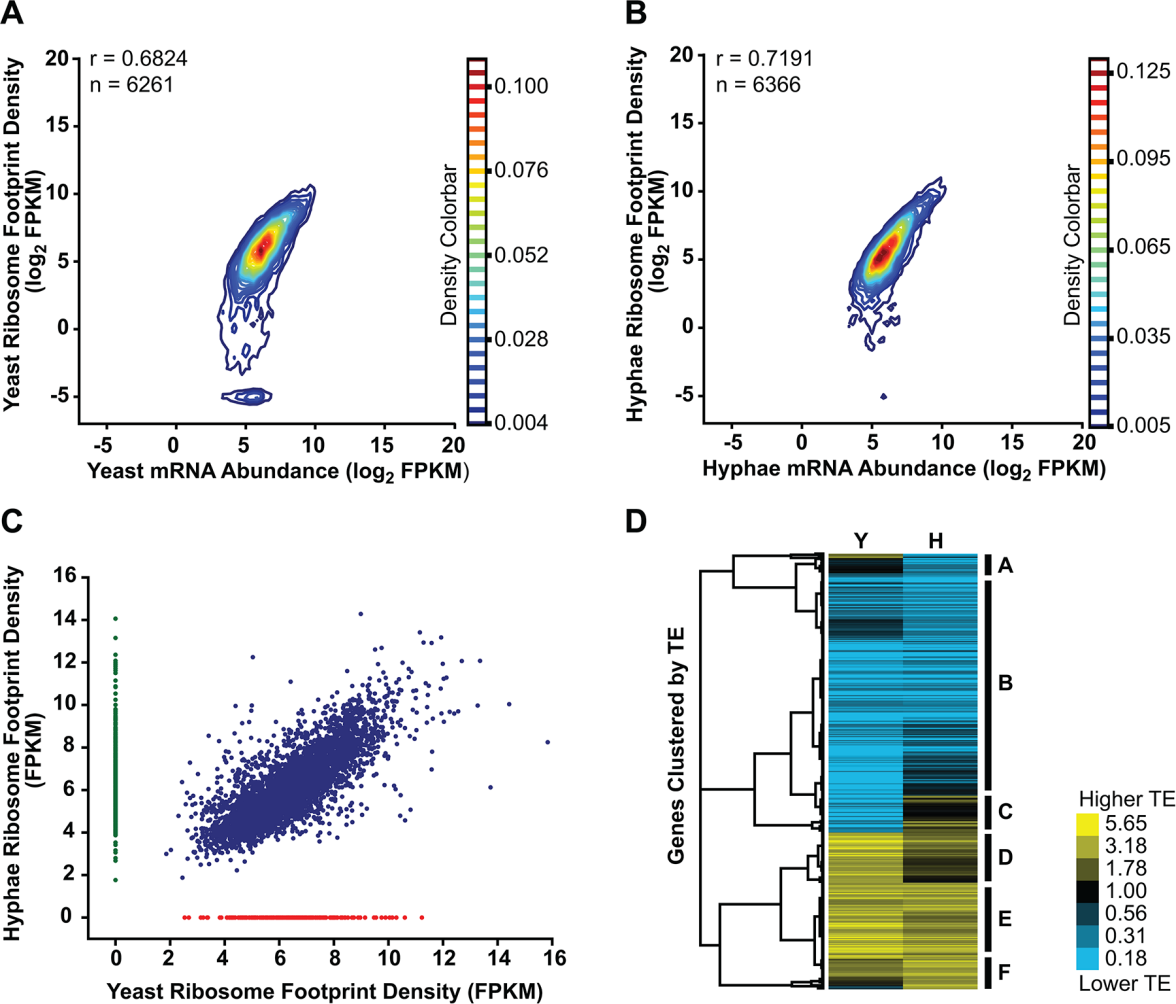
Transcriptional and translational regulation contributes to gene expression in *Histoplasma*. (A–B) Contour densities of log_2_ ribosome footprint density and mRNA abundance FPKM values were plotted as Gaussian kernel density estimate distributions for yeast (A) and hyphae (B) for transcripts with raw mRNA counts in coding regions ≥ 128. For mRNA that passed this filter but had no measureable ribosome footprints, ribosome footprint FPKM values were clipped to the lower limit of log_2_ = -5. Ribosome footprint and mRNA biological replicate data were pooled. Pearson correlation coefficients (adjusted r values) are shown for genes where the footprint FPKM values did not have to be clipped to -5. (C) Ribosome footprint density measurements (FPKM) for yeast and hyphal transcripts with footprint counts ≥ 128 are plotted in blue. Transcripts with raw footprint counts ≥ 128 in one cell state and with differential counts of 5 fold or greater between cell states are plotted in red and green, respectively. These transcript sets represent transcripts predominately translated only in yeast (red) or hyphal (green) cell types. (D) Translationally regulated transcripts were defined as having TE measurements that were differential by log_2_ 1.25 in at least one *Hc* cell morphology. Transcript TE values meeting this cutoff were then subjected to hierarchical clustering using an uncentered Pearson correlation. Data are displayed as a heatmap of log_2_ differential FPKM values with yellow representing genes with higher TE values and blue representing genes with lower TE values. Categories A-F are described in the text. Clustered heatmaps are given as S28–S29 Data.

Transcriptome-wide ribosome occupancy measurements did not correlate perfectly with mRNA abundance in *Hc* yeast or hyphae (**Fig 6A and 6B**). This suggested that expression of a subset of *Hc* genes is controlled at the level of translation and thus could reflect cell-type specific translational regulation between *Hc* cell morphologies. To investigate this, we first examined ribosome footprint density measurements to identify transcripts with different levels of translation between yeast and hyphal cells. Through this analysis we found subsets of transcripts that were predominantly translated only in one cell type (yeast = 200 transcripts and hyphae = 490 transcripts; **Fig 6C**; **S27 Data**) and reasoned that these transcripts encoded proteins with biological functions that are specific to a given cell type.

Since protein abundance in a cell is influenced by both transcript levels as well as the rate at which transcripts are translated, we also examined the translational efficiency of transcripts in yeast and hyphal cells. Clustering of yeast and hyphal TE values for transcripts that exhibited translational regulation highlighted at least 6 categories of translational regulation between yeast and hyphae (**Fig 6D**; **S28–S29 Data**). Most noticeably, we identified a large class of genes with low translational efficiency in both yeast and hyphae (**Fig 6D**- **Category B)**; not surprisingly many transcripts in this category encode proteins that are not expected to serve a function in *Hc* vegetative yeast or hyphae under the growth conditions used in these experiments. For example, genes predicted by homology to be involved in mating/meiosis (*HOP1*, *MEI2*, *STE2*, *STE6*), light sensing during circadian rhythm (*WHC2*), as well as conidial (asexual spore) development and biology (*CATA*, *RDS1*, *CON132*) showed low levels of translational efficiency in both yeast and hyphal cells. We also found many transcripts with robust translational efficiency in both cell types (**Fig 6D**- **Category E**) that included genes involved in routine cellular functions such as fatty acid biosynthesis (*ACC1*, *FOX2*, *FAA1*), cytoskeletal organization (*ACT1*, *BUD6*, *ARP1*, *SLA1*), and nuclear and mitochondrial transport (*KAP95*, *TOM20*, *KAP108*, *TIM16*). Categories where we observed translational efficiency differences between yeast and hyphae (**Fig 6D**- **Category A, C, D, F**) included some previously identified yeast and hyphal-enriched transcripts, indicating that a subset of cell-type enriched transcripts are regulated at both the transcriptional and translational level by the temperature-dependent developmental program.

### A subset of longer leader transcripts exhibit altered translational outcomes

**Fig 7:**
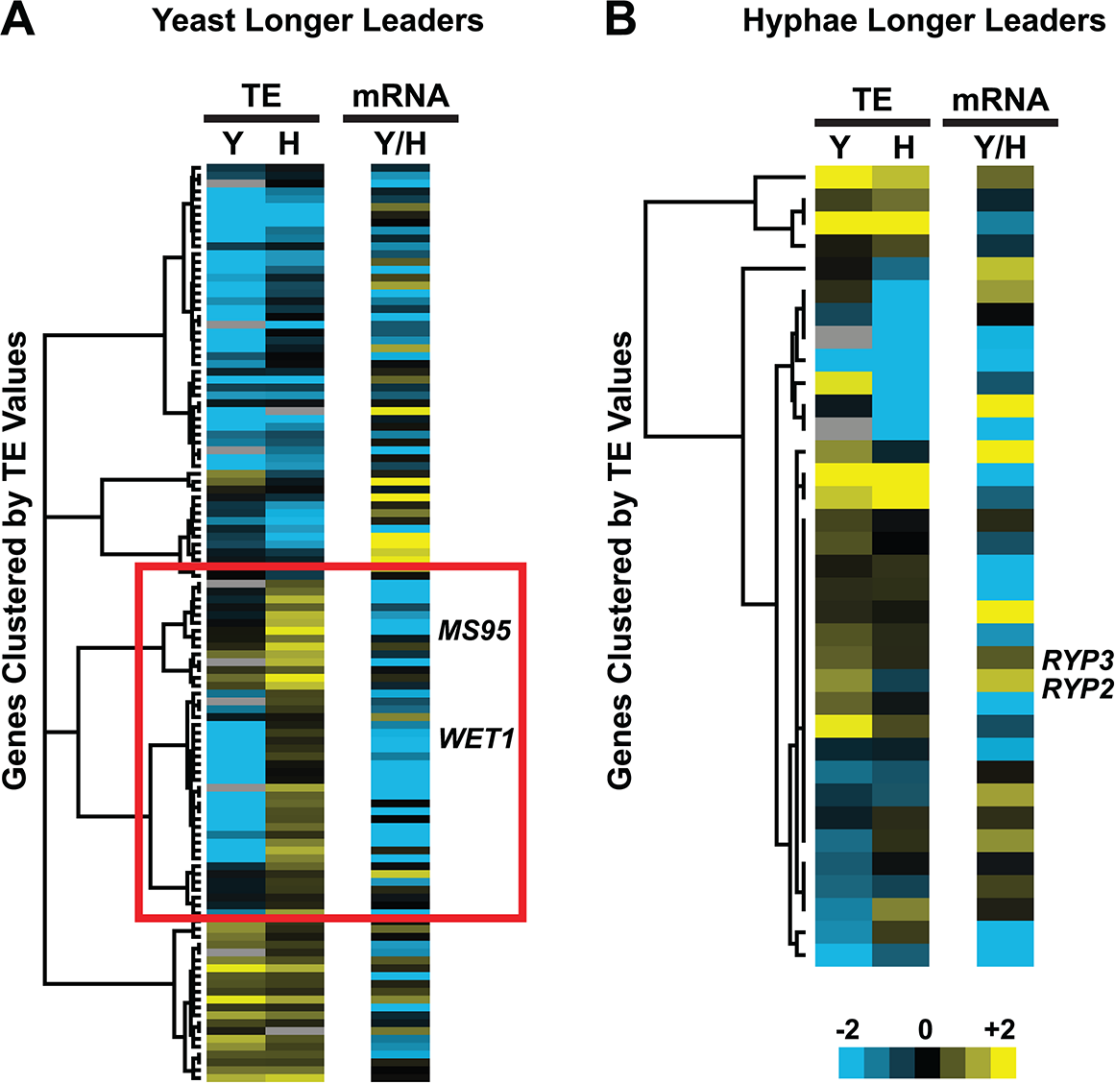
Many yeast-phase longer leader transcripts exhibit transcriptional and translational regulation. Yeast and hyphal phase TE values for G217B yeast-phase (A) and hyphal-phase (B) longer leader transcripts with TE values above the counting error threshold of 128 in at least one state (n = 116/120 transcripts for yeast n = 34/35 transcripts for hyphae). Transcript TE values were subjected to hierarchical clustering using an uncentered Pearson correlation. Data are displayed as a heatmap with yellow representing genes with higher TE values, blue representing genes with lower TE values, and grey representing genes that did not meet the counting error threshold. The corresponding Y/H mRNA expression values are shown alongside the clustered TE values with yellow representing transcripts upregulated in yeast cells and blue representing transcripts upregulated in hyphae. The yeast longer leader transcripts boxed in red indicated clusters where many transcripts are transcribed at a lower level and have a lower TE in yeast relative to hyphae. Particular genes called out in the text (*MS95*, *WET1*, *RYP3*, and *RYP2*) are labeled. Clustered heatmaps are given as S30–S33 Data.

**Fig 8:**
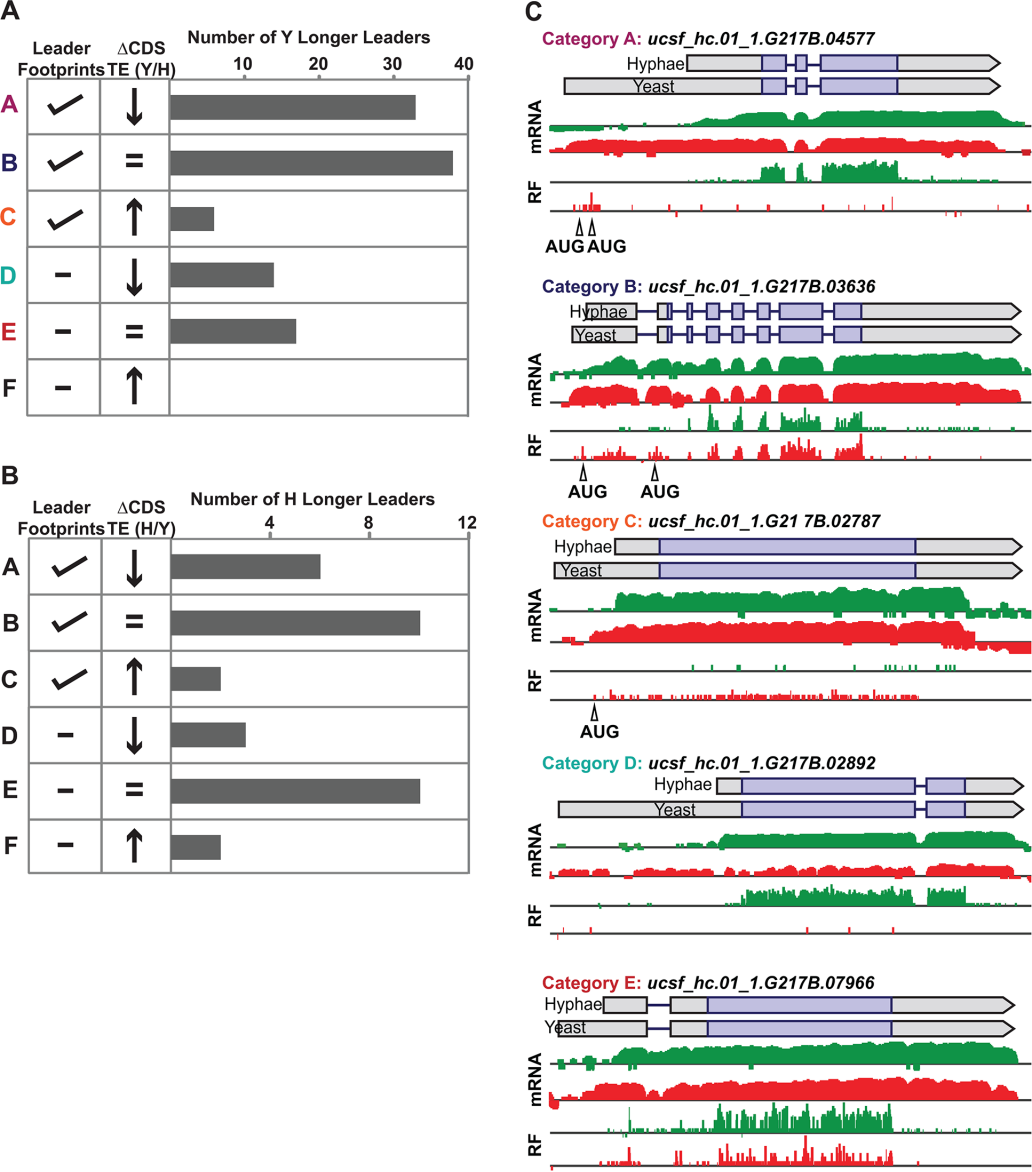
Ribosome footprint density patterns suggest categories of translational regulation for longer leader transcripts. (A–B) Categorization of yeast (A) and hyphal (B) longer leader transcripts with respect to their ribosome footprint density patterns. Yeast and hyphal longer leader transcripts were grouped into patterns based on longer leader region ribosome occupancy and CDS TE values. Categories were determined as follows: a: Ribosome density on longer leader region and decreased differential TE for CDS of longer leader transcript, b: Ribosome density on longer leader region and no change in TE for CDS of longer leader transcript, c: Ribosome density on longer leader region and increased differential TE for CDS of longer leader transcript, d: No ribosome density on longer leader region and decreased differential TE for CDS of longer leader transcript, e: No ribosome density on longer leader region and no change in TE for CDS of longer leader transcript, f: No ribosome density on longer leader region and increased differential TE for CDS of longer leader transcript. A change in CDS TE value of ≥ 2 fold was used to define a TE as differential in this figure. (C) Examples of ribosome density for yeast-phase longer leader transcript categories. Ribosome footprint (log_2_ RF) and corresponding mRNA (log_2_ mRNA) read coverage is plotted in red for yeast and green for hyphae. Schematics of the yeast and hyphal transcript structures are shown above the read coverage tracks with the predicted CDS regions highlighted in dark purple. Longer leader AUG start codons with ribosome density that are upstream of the predicted CDS are highlighted. Leader identities for each category are given in S27 Data.

Having established ribosome profiling as a method to measure ribosome occupancy and translational efficiency in *Hc* yeast and hyphae genome-wide, we next examined the translational outcomes of differential leader transcripts. Comparing yeast and hyphal TE values for the set of longer leader transcripts revealed that many exhibited altered translational regulation compared to their cognate shorter transcript isoforms (**Figs 7**and **S14**; **S30–S33 Data**). Correlation of ribosome footprint densities on differential leader regions with changes in TE values (by examining a 2 or 1.5 fold change in CDS TE) between short and long transcript forms resulted in classification of leaders into broad regulatory patterns (**Figs 8 and S15**). The most striking pattern we observed was differential leader ribosome density that correlated with a decrease in TE, reminiscent of uORF mechanisms of translational repression (**Fig 8**- **Category a**). We also observed longer leaders with no ribosome density and strong repression of downstream CDS translation (**Fig 8**- **Category d**) via unknown mechanisms. In addition, we found examples of robust leader ribosome occupancy that resulted in no change to the TE, which may represent cases of N-terminal protein extensions and uORFs that don’t influence translation of the CDS (**Fig 8**- **Category b**). Together these data suggest that as many as half of the observed longer leaders may be involved in translational repression of the associated CDS. The features distinguishing such regulatory leaders remain to be elucidated experimentally.

While examining longer leader transcripts that exhibited strong repression of CDS translation (**Fig 8**- **Category a**, **d**), we noticed that many of these transcripts had longer leaders in the yeast phase and were additionally regulated at the level of transcription between yeast and hyphae (**Fig 7A**). Specifically, a subset of yeast-phase longer leader transcripts were transcriptionally and translationally repressed in yeast cells, yet were robustly transcribed and translated in hyphae (**Fig 7A**- **red box**). To validate whether these ribosomal profiling data corresponded to levels of protein accumulation, we expressed *GFP* under the control of the *MS95* longer leader transcript promoter and leader region (*MS95*p) in *Hc* cells and examined GFP protein production at 37°C and RT. *MS95* has a longer leader transcript form in yeast cells that exhibits lower translational efficiency compared to its shorter hyphal form (**S16A Fig**). Assessment of GFP protein levels by Western blot in *Hc* cells expressing the *MS95*p – GFP construct and grown at 37°C or RT recapitulated the differential protein expression pattern we inferred from ribosome profiling (**S16B and S16C Fig**). Namely, the *MS95* upstream region restricts expression at 37°C, but allows robust transcription and translation at RT. Why *Hc* cells tightly restrict expression of Ms95 at 37°C is unclear as the biological function of Ms95, a homolog of the DNA damage and heat-stress responsive protein Ddr48 in *S*. *cerevisiae*, remains unknown.

### Dysregulation of a longer leader transcript influences *Histoplasma* cell fate

Much precedence exists for cells to employ translational regulation to achieve tight spatial or temporal control of protein expression during developmental processes [53]. In this vein, we noticed that some longer leader transcripts have been implicated in governing *Hc* cell fate decisions. Both *RYP2* and *RYP3*, master regulators of yeast cell morphology, have longer leader regions in hyphal cells. *RYP2* has ribosome density in its differential leader region and lower TE of the *RYP2* CDS in hyphae versus yeast (**S17A Fig**). The role of the *RYP3* hyphal longer leader region is unclear as there is only a slight reduction in TE of the *RYP3* CDS in hyphae versus yeast (**S17B Fig**). Ryp2 and Ryp3 both associate with DNA to regulate transcription of yeast-phase enriched transcripts at 37°C; furthermore, disruption of *RYP2* or *RYP3* in *Hc* cells results in inappropriate hyphal growth of *Hc* at 37°C [9,54]. Thus, tight restriction of Ryp levels during cellular development may be a mechanism used by *Hc* to control appropriate morphology in response to temperature.

**Fig 9:**
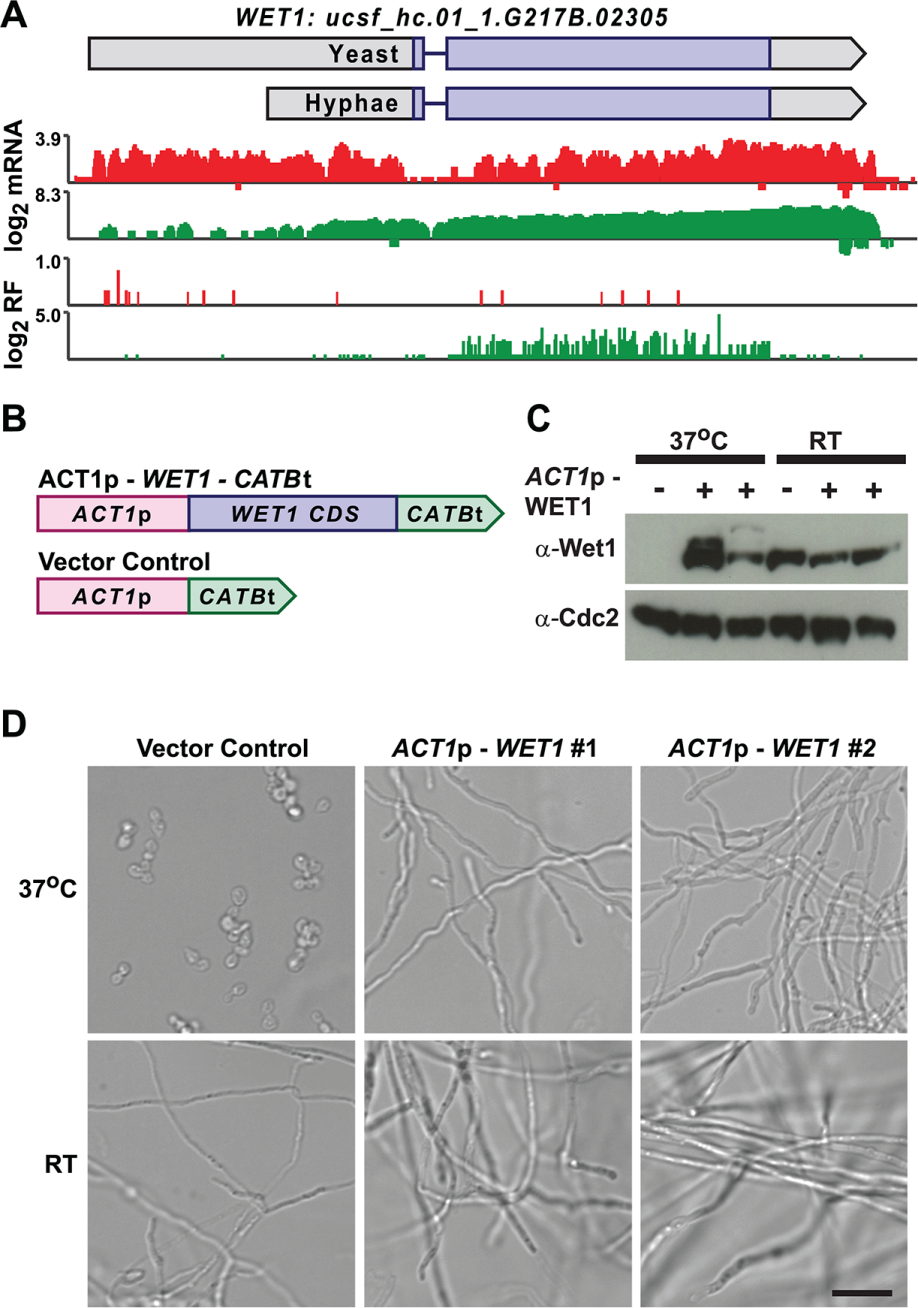
Misregulation of Wet1 expression induces inappropriate hyphal growth at 37°C. (A) Ribosome footprint and corresponding mRNA read coverage is shown for *WET1*. Log_2_ yeast read coverage is plotted in red and log_2_ hyphal read coverage is plotted in green. The *WET1* assembled transcript structures are indicated above the read coverage tracks with the predicted CDS region highlighted in dark purple. (B) Schematic of Wet1 mis-expression and vector control constructs. *ACT1*p, actin promoter. *CATB*t, catalase B terminator. (C) Western blot examining Wet1 total protein levels in cells transformed with the *ACT1*p –*WET1* –*CATB*t or vector control construct and grown at either 37°C or RT. (D) Confocal DIC microscopy showing cell morphology of vector control and *ACT1*p –*WET1* –*CATB*t transformed cells grown at 37°C or RT. Scale bar, 20 μm.

We mined *Hc* longer leader transcripts for additional candidates that could be involved in regulating cell fate decisions by identifying longer leader transcripts with transcription factor domains or homology to developmental regulators in other fungal species. This approach identified 14 putative developmental regulators with longer leader regions in *Hc* yeast or hyphal cells (**S3 Table**). One putative *Hc* developmental regulator we identified was *WET1*, which we named based on its homology to the *Aspergillus nidulans* regulator of conidial (asexual spore) development, WetA [13,55]. *WET1* has a longer leader sequence in *Hc* yeast cells and its CDS region is translationally repressed in yeast while the shorter hyphal transcript form has robust ribosome CDS occupancy in hyphae (**Fig 9A**). To explore the idea that regulators of *Hc* developmental programs include longer leader transcripts that are tightly regulated between yeast and hyphal cell types, we placed the CDS of *WET1* under the control of a heterologous *Hc* promoter, *ACT1*p (**Fig 9B**). Importantly *ACT1* is not differentially translationally or transcriptionally regulated between *Hc* yeast and hyphae. Ectopic expression of this *ACT1*p – *WET1* construct in *Hc* yeast cells resulted in inappropriate hyphal growth of *Hc* at 37°C (as compared to vector control cells; **Fig 9C and 9D**), while expression of the *ACT1*p – *WET1* construct had no discernible effect on *Hc* hyphal morphology at RT (as compared to vector control cells; **Fig 9D**). These data indicate that restricting Wet1 expression in yeast-phase cells is critical for maintenance of the yeast cell developmental program and that *Hc* longer leader transcripts are interesting candidates for *Hc* developmental regulators.

## Discussion

Here we present improved transcript models for multiple clinical *Histoplasma* isolates as well as the first look at the translational landscape of this medically relevant human fungal pathogen. Through this work we find that *Hc* alters transcript leader length between its morphologic cell types for a subset of its transcriptome. Transcript leader length is a biologically regulated outcome of transcription in *Hc* as our work uncovers many examples of leader extensions (187 transcripts) that are evolutionarily conserved across *Hc* phylogenetic lineages. Ribosome and mRNA density measurements of longer leader transcripts reveal a class of genes that are under tight translational and transcriptional regulation. Further examination of this group of transcriptionally and translationally regulated genes indicated that some are involved in controlling *Hc* morphology and that their strict regulation may be necessary for appropriate developmental decisions.

Our ribosome profiling experiments suggested mechanisms of translational regulation for longer leader transcript CDS regions. Namely, we observed cases where ribosome density on longer leader regions correlated with reduced translational efficiency of the downstream CDS, suggesting that this class of transcripts may be translationally repressed via well-studied mechanisms of uORF-mediated translational control [56]. Alternatively, we observed cases of translationally repressed CDS regions of longer leader transcripts with no evidence of ribosome occupancy on the leader, indicating that translational repression may be regulated via uORF-independent mechanisms. Future work will be needed to examine mechanisms of translational repression for this class of transcripts such as changes to mRNA secondary structure of the leader that inhibit ribosome scanning or cell-type specific factors that regulate ribosome function. For example, leader sequences could specify subcellular localization of mRNAs, which could inhibit (or facilitate) translation. Lastly, we identified longer leader transcripts that exhibited ribosome footprint density in the leader region directly upstream of the predicted CDS with little effect on translational efficiency. We suspect that some of these longer leader transcripts could serve to encode alternate protein variants and thus represent cases where leader extensions increase proteome diversity. We must also emphasize that some of our longer leader transcripts appear to be part of a complex mixture of transcript isoforms of varying sizes (as seen by Northern blot; see S11 Fig), which will complicate future mechanistic analyses.

The mechanism by which differential leader sequences are specified by distinct cellular states is yet to be determined. One of several possibilities is that a particular transcript initiation site may be occluded in one state by protein complexes that associate with the DNA. Intriguingly, we observed that 13 of the 49 transcripts with a longer leader sequence in the hyphal phase (including Ryp2; see **S17A Fig**) show association of the corresponding DNA with a yeast-enriched protein complex (p = .0015, given 785 total Ryp associated transcripts). Specifically, in the yeast-form at 37°C, these genes are bound by the Ryp proteins [9], which are yeast-enriched transcription factors that are required for yeast-phase development. It is possible that the physical presence of the Ryp complex on the DNA at 37°C could interfere with production of the longer-leader transcript; consequently the longer transcript isoform appears only in hyphal cells at room temperature. This model is appealing because it ties the presence of state-specific transcription factors to state-specific transcript architecture.

It is accepted that metazoans employ translational regulation to precisely tune gene expression during developmental processes [53]. Additionally, pathogens can regulate virulence factors at the translational level using host cues, such as temperature, to induce virulence factor expression once in the host environment [57,58]. Thus, translational control is a mechanism that can be used to quickly or precisely regulate gene expression during cellular development and pathogenesis. However, the biological function of many of the translationally regulated longer leader transcripts in *Hc* is unknown, making the context and significance of their translational regulation difficult to deduce at this time. We hypothesize that longer leader transcripts that are translationally repressed in the yeast phase could play a role in regulating hyphal growth or promoting morphogenesis in response to a change in temperature from 37°C to RT. Repression of such genes during yeast-phase growth would be critical to prevent inappropriate hyphal growth, which we have demonstrated for the longer leader transcript *WET1*. Alternatively, longer leader transcripts that are translationally repressed in hyphae could be tightly regulated for appropriate expression during *Hc* pathogenesis (since yeast cells are the disease-causing host form of *Hc*). Ultimately, a better understanding the biological function of translationally regulated longer leader transcripts will inform ideas for how *Hc* cells use translation (or appear to use changes in transcript structure) to regulate expression of a subset of the transcriptome.

In addition to uncovering examples of extended leader transcripts that exhibit distinct modes of translational regulation, our work provides a better foundation for understanding the biology and pathogenesis of the human pathogen *Histoplasma*. For example, our discovery of an expansion of knottin family proteins was enabled by the identification of small transcripts that show conserved enriched expression in the parasitic phase of this organism. Additionally, as with many organisms, it is becoming apparent that the regulation of *Hc* gene expression and transcript architecture is more complex and nuanced than previously appreciated. For example, our work demonstrates that both transcriptional and translational regulation contribute to the yeast and hyphal developmental programs. By deciphering the transcript structures, transcript expression patterns, as well as identifying novel orthogroups that likely encode short peptide products and regulatory RNAs across *Hc* lineages, we have laid the groundwork for a deeper understanding of the *Hc* strains that cause a wide variety of disease complications in the human host.

## Materials and Methods

### Strains and culture conditions

*Histoplasma capsulatum* (*Hc*) strains G217B (var. *capsulatum*; ATCC 26032), G217B*ura5*Δ (var. *capsulatum*; WU15), G186AR (var. *capsulatum*; ATCC 26029) were all gifts from the laboratory of William Goldman, University of North Carolina, Chapel Hill. H88 (var. *duboisii*; ATCC 32281) and H143 (var. *capsulatum*; CBS 287.54) were obtained from the American Type Culture Collection and Centraalbureau voor Schimmelcultures, respectively. *Hc* strains were routinely grown in HMM (Histoplasma-macrophage medium) broth or plates [59] or Sabouraud dextrose (Difco, BD, San Jose, CA) agar plates. Media was supplemented with 200 μg/mL of uracil (Sigma-Aldrich, St. Louis, MO) where indicated. *Hc* cultures were grown at 37°C under 5% CO_2_ for yeast-phase growth or at room temperature (RT) for hyphal-phase growth with continuous shaking of liquid cultures on an orbital shaker.

### Paired-end RNA sequencing

G217B, G186AR, H88, and H143 yeast cells were inoculated from HMM agarose plates into HMM liquid medium for yeast-phase growth. Yeast cells were passaged at a 1:25 dilution three times and 2-day cultures of each strain were harvested for RNA collection after the third passage. For hyphal cells, G217B, G186AR, H88, and H143 hyphae were inoculated from Sabouraud dextrose agar plates grown at RT into HMM liquid medium. Hyphal cells were grown for 4–6 weeks with passaging 3 times (1:5 dilution) into fresh HMM medium at RT before reaching a sufficient density of cells for harvesting. Hyphal and yeast cells were collected by centrifugation or filtration and total RNA was isolated using a guanidine thiocyanate lysis protocol as previously described [13]. Paired-end RNA-seq libraries were made from biological duplicate cultures of G217B, G186AR, H88, and H143 for both yeast and hyphae. DNA was depleted from 10–20 μg of total RNA by 1 hour of DNaseI digestion at 37°C (Ambion by Life Technologies, Carlsbad, CA). Poly(A)+ RNA was selected from DNA-depleted RNA on oligo-dT25 DynaBeads (Invitrogen by Life Technologies) using 2 rounds of selection per the manufacturer’s instructions. Approximately 50 ng of poly(A)+ RNA was used as input for generating sequencing libraries with the ScriptSeq v2 RNA-Seq Library Preparation Kit (Epicentre, Madison, WI) per the manufacturer’s instructions with the following modifications. After 11 cycles of the ScriptSeq v2 PCR cDNA amplification step, cDNA libraries were resolved on an 8% TBE gel (Invitrogen by Life Technologies) and a 350–500 bp range of cDNA library was excised, eluted, and precipitated from the gel. Seven additional PCR cycles were resumed following the ScriptSeq v2 Kit protocol. cDNA libraries were purified with AMPure XP beads (Beckman Coulter, Brea, CA) to deplete primers. Libraries were multiplexed and subjected to 100 bp paired-end sequencing using the Illumina HiSeq2000 sequencer (Illumina, San Diego, CA).

### Sources of *Histoplasma* genome assembly and annotation

Throughout this work, the following sources of *Hc* genomic sequences and gene predictions were used: version 2 of the H88, H143, and G168AR genome assemblies and predicted gene sets from the BROAD Institute, retrieved on 6/15/2011 from http://www.broadinstitute.org/annotation/genome/dimorph_collab/MultiDownloads.html and the 11/30/2004 version of the G217B genome assembly and 9/21/2005 predicted gene set from the Genome Sequencing Center at Washington University (GSC) as mirrored at http://histo.ucsf.edu/downloads/.

### Genome-guided transcriptome assembly from paired-end reads

We used a genome-guided approach to assemble *Hc* transcriptomes independently for each of the 4 *Hc* strains from our paired-end sequencing data. The bulk of our analysis was based on combining reads from all yeast and hyphal biological replicates in a given strain. For the longer leader analysis, we also assembled state-specific transcriptomes using only yeast or hyphal reads. In all cases, the same assembly pipeline was applied, as outlined in **Fig 2A** and described below.

Paired-end reads were pre-processed to remove high copy sequences that were not of interest; specifically, all read pairs were searched against the mitochondrial genome, a representative full length MAGGY transposon, and the 45S rDNA locus using bowtie2 version 2.0.0-beta7 [60] with a minimum fragment length (-I) of 50 and a maximum fragment length (-X) of 800. Read pairs that did not align to the above sequences were aligned to the appropriate genome assembly (GSC assembly for G217B, BROAD assembly for G186AR, H88, and H143) using GSNAP [61] with parameters based on the alignReads.pl script from TRINITY [62] (allowing novel splice sites (-N 1), up to 20 alignments per pair (-n 20), and up to 10 kb introns (-w 10000)). Transcribed fragments (transfrags) were initially defined as contiguous genomic regions with GSNAP-based read coverage > = 8 reads/base. Initial transcripts were then assembled by joining transfrags separated by no more than 2300 bases and spanned by at least 6 single reads or read pairs. Introns were annotated as GSNAP-identified splice sites falling within the initial transcripts. Where multiple splice sites spanned the same genomic location, only the splice site supported by the greatest number of aligned reads was annotated as an intron. Transcripts were identified as antisense artifacts and removed if they met either of the following criteria: 1) all splice junctions in the transcript were CT-AC (antisense to the canonical GT-AG); 2) the transcript was completely spanned by a higher abundance transcript on the opposite strand and contained either no introns or at least one non-canonical splice junction. Initial coding sequences (CDS) were then annotated as the largest open reading frame (ORF) in each transcript, with a minimum ORF size of 60 codons. This initial assembled transcript set contained fusions due to adjacent, same-strand transcripts separated by intergenic regions that were small or non-existent (e.g., due to overlapping 3' and 5' ends). We addressed this assembly artifact by identifying initial transcripts spanning multiple gene predictions. Specifically, we identified all ORFs greater than 60 bp within each initial transcript, then looked for same-strand overlaps between these ORFs and the upstream predicted protein sets (from the GSC or the BROAD); where an initial transcript contained ORFs that mapped independently to at least two different sets of predicted proteins, such that an untranslated subsequence of the transcript divided the two sets without overlapping either, the transcript was split at the midpoint of the dividing subsequence, and the CDS of the resulting transcripts were reannotated as the largest ORF of at least 60 bp.

For the pooled transcriptome assembly, transcripts were named as ucsf_hc.01_1.(strain).(gene_id), where strain is G217B, G186AR, H88, or H143 and gene_id is a five digit, zero padded integer. For the state-specific transcriptome assemblies, transcripts were named as ucsf_hc.01_1.(strain)(state).(gene_id), where state is Y or H for yeast and hyphae, respectively, and the remaining fields are as above. gene_id is unique within a given transcriptome assembly, and there is no deliberate correspondence among gene_id values from different assemblies.

### Defining orthogroups and ortholog pairs across *Histoplasma* strains

For ortholog assignment by InParanoid [19], InParanoid version 1.35 was run with default parameters and no outgroup for each pair of *Hc* strains, using either predicted or assembled protein sequences as inputs.

For orthologroup assignment by Mercator [18], Mercator version 0.4 (compiled from commit 991c85a of the cndsrc git repository: http://www.biostat.wisc.edu/~cdewey/software/cndsrc.git) was run in draft mode on the 4 *Hc* genome assemblies using all-against-all BLASTP searches of the assembled protein sets as anchors. This resulted in 5509 complete orthogroups (containing one gene from each strain) and 2356 incomplete orthogroups. In order to find orthologous genes present in the transcript assemblies but missing from the BLASTP searches due to inappropriate CDS annotations, protein sequences from incomplete orthologs were searched against the genome assemblies of missing strains using TBLASTN [20] with soft masking and an expect threshold of 1e-6, then finding the assembled transcript with greatest same-strand overlap to the top TBLASTN hit; transcripts detected this way were added to the existing orthogroups, as long as TBLASTN searches with each member of the orthogroup did not detect different transcripts and multiple orthogroups did not detect the same transcript. Application of this TBLASTN-based strategy resulted in an additional 1282 complete ortholog groups, for a total of 6791.

BLAST runs for both InParanoid and Mercator were carried out with version 2.2.26 of NCBI BLAST

### Quantification of mRNA expression from paired-end reads

Relative abundances (reported as FPKM values [63,64]) for each transcript in each sample were estimated by aligning read pairs to the transcriptome assembly for the corresponding genome with bowtie2 version 2.0.0-beta7 [60] with a maximum fragment length (-X) of 800, allowing all alignments (-a), and submitting the output to eXpress version 1.3.1 [25] specifying strand-specific read pairs (—fr-stranded). Transcripts with FPKM values ≤ 1.0 in yeast or hyphae were clipped to a value of 0. FPKM Y/H values were median normalized per strain using transcripts with FPKM values ≥ 10.0

### Protein annotation

Assembled CDS were annotated with Pfam domains by searching all protein sequences against version 27.0 of Pfam-A [21] with hmmscan from HMMer 3.0 (http://hmmer.org/). A Pfam domain was considered to be conserved if it was matched by at least 3 ORFs in a given orthogroup.

Assembled CDS were annotated as secreted if a signal peptide was detected in the corresponding protein sequence by Phobius version 1.01 [23].

### Knottin phylogeny

The curated Fungi1 alignment was downloaded from the KNOTTIN database [34] and trimmed to the conserved cysteine residues plus 10 residues of padding on either side (alignment positions 423 through 483, counting from 1). Hmmer 3.0 (http://hmmer.org) was used to build an HMM from the trimmed alignment and to search the resulting model against the *Hc* assembled transcriptomes, predicted gene sets for 41 fungal genomes (see **S18 Data**), and the curated Fungi1 sequences using an expect threshold of 1 e-2, yielding 167 total hits, 121 of which corresponded to unique (non-redundant) genes. Non-redundant hits were aligned to the HMM with hmmalign, and a phylogenetic tree was inferred from the aligned positions using fasttree2 [65]. The phylogenetic tree is available as supplementary file 16 and the protein alignment is available as S17 Data.

To generate the tree in Fig 3B, the full tree was pruned to the *Hc* G217B assembled transcripts plus additional sequences representative of the diversity of the G217B-containing clades, maintaining the topology and branch lengths of the full tree. To generate the reduced alignment shown in Fig 3C, the full alignment was reduced to just the *Hc* G217B assembled transcripts and *AVR9*, and adjusted with the following manual improvements: removing gap-only columns, removing staggered gaps at the N-terminus to improve the alignment of the aliphatic/aromatic position at +3 relative to the first cysteine, removing gaps at the C-terminus of ucsf_hc.01_1.G217B.08018 to align the final cysteine, and trimming all sequences to the aligned AVR9 positions.

### Generation of ribosome footprint libraries

Biological duplicates of G217B yeast cells were grown at 37°C in HMM liquid medium to mid-log phase. Biological duplicates of G217B hyphal cells were grown for 4–6 weeks with passaging three times (1:5 dilution) into fresh HMM medium at RT before reaching a sufficient density of cells for harvesting. Hyphal and yeast cells were treated with 100 μg/mL cycloheximide for 2 minutes (MP Biomedicals, Santa Ana, CA) before harvesting of cells by filtration. Total RNA for mRNA library preparation was isolated from a small fraction of the total yeast or hyphal cells collected for ribosome profiling using a guanidine thiocyanate lysis protocol as previously described [13]. Ribosome profiling and matched mRNA-seq sample preparation and library building were performed as described [49], except that the 3′ linker ligation strategy was used instead of poly(A) tailing for marking and capturing the 3’ RNA end [50]. Libraries were multiplexed and subjected to 50 bp single-end sequencing with an Illumina HiSeq2000 sequencer (Illumina).

### Mapping and quantification of ribosome footprint and corresponding mRNA reads

The strand-specific, single-end reads from the matched mRNA and ribosome footprint samples were processed identically, except as noted. Due to the relatively short lengths of these reads, alignment steps were performed with bowtie version 0.12.7 [66], rather than the bowtie2/GSNAP approach used for the paired-end data.

Reads were pre-processed by stripping 3' sequence matching the primer linker sequence, allowing 20% mismatches, filtering any reads that were less than 11 bp after linker stripping, and additionally filtering for matches to the mitochondrial, MAGGY, and rDNA sequences described above (see paired-end read assembly) using bowtie with default parameters.

The remaining, linker-stripped sequences were mapped to the genome with bowtie, restricting the output to unique alignments (-k1) and post-filtered for full length alignments of the query sequences. We noted a strong bias for T mismatches in the first aligned position, consistent with previously observed terminal nucleotidyl transferase activity of reverse transcriptase [67]; to address this, we removed all first position T mismatches, treating the second aligned position as the true 5' of the sampled fragment for the remaining steps. Other than this special treatment of the first position, all alignments were required to be perfect matches between query and genome sequence. Aligned sequences were required to be at least 22 bp and, for ribosome footprint samples only, no more than 32 bp.

For alignments passing the above criteria, the 13th aligned position, inferred to correspond to the ribosomal P-site for ribosome footprint samples, was taken as the location of the mapped read.

Assembled transcript FPKMs for each sample were calculated as the number of reads located in the CDS of that transcript, divided by the length of that CDS in kilobases and the total number of CDS-mapped reads for the sample in millions. The same formula was used for quantifying ribosome footprints in leader regions, defining the leader as all transcript sequence 5' of the first CDS position, and normalizing by the length of the leader and the total number of CDS-mapped reads (to allow direct comparison of CDS and leader FPKMs).

### Identifying longer leader transcripts

Yeast and hyphae per-state assembled transcript calls were used to identify transcripts with differential leader regions. Leader regions were calculated as the distance from the start of the 5’ transcript end to the beginning of the predicted CDS. Leaders were defined as differential between *Hc* cell types if the change in size of the yeast and hyphal leader lengths was measured to be ≥ 100 bp in 3 out of 4 *Hc* strains in the per-state transcript assemblies. Transcripts meeting these criteria were further evaluated manually to determine whether the observed mapped read density supported a 5’ transcript extension in one cell type. For assessment of translational efficiency and ribosome occupancy of longer leader transcripts, the set of conserved longer leader transcripts were further manually evaluated against the G217B mRNA read coverage from the ribosome profiling experiments to ensure that the observed mapped read density supported a 5’ transcript extension in one cell type.

### Clustering

Cluster 3.0 was used to perform hierarchical clustering of genes using an uncentered Pearson correlation [68,69]. Clustered data was visualized using Java Treeview 1.1.4r4 (available at http://jtreeview.sourceforge.net) [70].

### *MS95*p –*GFP* time course

2781 bp of the *MS95* promoter/leader region (*MS95*p), 1045 bp of the *GAPDH* promoter (*GAPDH*p), and 729 bp of the *CATB* terminator (*CATB*t) were amplified from G217B gDNA and assembled into a Gateway entry vector pDONR/Zeo (Life Technologies) containing enhanced GFP (e*GFP*) using a CPEC cloning strategy [71]. This generated BAS1464 (*MS95*p –e*GFP–CATB*t) and BAS1514 (*GAPDH*p–e*GFP*–*CATB*t) constructs. All primer sequences are included in **S4 Table.** Using LR Gateway cloning (Life Technologies) each pDONR/Zeo entry vector was recombined into the *Hc* episomal expression vector pDG33 (pDG33 is a derivative of pWU55 [72] with *Hc URA5* added for selection and made Gateway compatible). The episomally-maintained positive control (*GAPDH*p–e*GFP*–*CATB*t) and *MS95*p –e*GFP–CATB*t constructs were electroporated into G217B*ura5*Δ as previously described [72].

G217B*ura5*Δ strains transformed with the *GAPDH*p and *MS95*p eGFP constructs were grown at 37°C to late log phase, diluted 1:25 into 5 mL HMM medium for growth at 37°C or 1:10 into 10 mL HMM medium for growth at RT. At 1 d, 2 d, and 3 d post-inoculation, cells were harvested by centrifugation and protein and RNA was isolated simultaneously from each 37°C or RT culture using Qiazol (Qiagen, Netherlands) following the manufacturer’s instructions.

### Ectopic expression of *WET1*

The *Hc WET1* coding sequence, 1035 bp of the *ACT1* promoter (*ACT1*p), and 729 bp of the *CATB* terminator (*CATB*t) were amplified from G217B gDNA and assembled into the Gateway entry vector pDONR/Zeo (Life Technologies) using restriction enzymes to generate BAS1504. A vector control construct, BAS252, was generated identically except lacking the *WET1* CDS. Using LR Gateway cloning (Life Technologies) each pDONR/Zeo entry vector was recombined into the *Hc* episomal expression vector pDG33 (pDG33 is a derivative of pWU55 [72] with *Hc URA5* added for selection and made Gateway compatible). The episomally-maintained vector control (*ACT1*p –*CATB*t) and *ACT1*p –*WET1 –CATB*t constructs were electroporated into G217B*ura5*Δ as previously described [72]. Protein and RNA was isolated simultaneously from each 37°C or RT culture using Qiazol (Qiagen, Netherlands) following the manufacturer’s instructions.

Cell morphology of vector control and *WET1*-expressing cells was determined using differential interference contrast (DIC) microscopy with a Yokogawa CSU-X1 (Yokogawa, Tokyo, Japan) spinning disk confocal mounted on a Nikon Eclipse Ti inverted microscope (Nikon, Tokyo, Japan) with a PLAN APO 40X objective (Nikon) and an Andor Clara digital camera (Andor, Belfast UK). Images were acquired by and processed in NIS-Elements software 4.10 (Nikon).

### Western blotting

10–20 μg of protein was resolved on a 4–12% NuPAGE Bis-Tris gel (Life Technologies) in MOPS buffer. For detecting endogenous levels of Wet1, a rabbit polyclonal peptide antibody was raised (Covance, Princeton, NJ) against Wet1 (epitope: KTKARREQEAREKRRKLS; ID: CA2890). GFP was detected using a mouse anti-GFP antibody (Roche Applied Bioscience, Indianapolis, IN; 11814460001). Equivalency of protein levels between samples was assessed with a rabbit antibody against Cdc2 (Santa Cruz Biotechnology, Santa Cruz, CA; sc-53).

### Northern blotting

5–10 μg of total RNA was separated on a 1.5% denaturing agarose-formaldehyde gel and transferred to a positively charged nylon membrane (Roche Applied Bioscience or PerkinElmer, Waltham, MA). Northern probes were generated with gene specific primers by amplifying ~ 200–500 bp of transcript from genomic DNA. Primer sequences are given in **S4 Table**. 100–150 ng of each DNA probe was labeled using the High Prime DNA Labeling Kit (Roche Applied Bioscience) and [α – ^32^P] dCTP (PerkinElmer). Membranes were blocked in UltraHybe hybridization buffer (Ambion, Life Technologies) for at least 30 minutes and denatured probe was added to the same blocking buffer and incubated at 42°C overnight. The next day membranes were washed twice in 2X SSC, 0.1% SDS for 5 minutes and twice in 0.1X SSC, 0.1% SDS for 15 minutes at 42°C before exposure to a phosphorimager screen (GE Life Sciences, Pittsburgh, PA). Screens were scanned with a Fuji FLA-5100 imager and analyzed with Multi Gauge Software (ver. 3.1; Fujifilm/GE Life Sciences).

### Categorization of ribosome density patterns on longer leader transcripts

Patterns of ribosome occupancy on G217B yeast and hyphal longer leader transcripts were used to determine categories of translational regulation. Categories were defined as follows: a: Ribosome density on longer leader region and decreased differential TE for CDS of longer leader transcript, b: Ribosome density on longer leader region and no change in TE for CDS of longer leader transcript, c: Ribosome density on longer leader region and increased TE for CDS of longer leader transcript, d: No ribosome density on longer leader region and decreased TE for CDS of longer leader transcript, e: No ribosome density on longer leader region and no change in TE of CDS for longer leader transcript, f: No ribosome density on longer leader region and increased TE for CDS of longer leader transcript. g: Ribosome profiling reads used to calculate TE CDS values are below the limit of accurate measurement (< 128 counts) in at least one cell type. We examined these categories using two different fold cut-offs for CDS TE values: a change in TE was defined as ≥ 1.5 fold (shown in S15 Fig) or ≥ 2 fold (shown in Fig 8). FPKM values and raw counts of ribosome density on leader regions were used to assist in determining the presence of ribosome density in differential leader regions after manual examination of the location of the footprint density. After manual confirmation of the location of footprint reads on each leader region, leaders with greater than 10 footprint reads in the differential leader region were considered to have ribosome density.

### Other software and libraries

MochiView [73] and IGV [74] were used to visualize sequencing reads. All statistical calculations were carried out in R 2.15.1 [75]. We wrote custom scripts and generated plots in Python 2.7, using the following open-source libraries: NumPy 1.6.2 and SciPy 0.10.1 [76], NetworkX 1.7rc1 [77], and Matplotlib 1.1.1rc2 [78]. IPython notebooks were used for interactive data exploration and collaboration [79].

### Transcriptome assembly gff3 files/GEO accession

Transcriptome assembly data for each *Hc* strain (G217B, G186AR, H88, and H143) are available as genomic features gff3 files (generic feature format version 3) (S1–S4 Data; S19–S26 Data). For high-throughput sequencing data, the raw data are available at the NCBI Sequence Read Archive (SRA) and Gene Expression Omnibus (GEO) databases [80,81] under GEO SuperSeries accession GSE68707.

## Supporting Information

S1 Fig *Histoplasma* assembled transcripts are longer in length than the *ab initio* predicted transcript models. The normalized kernel density estimate distributions for the length of all assembled transcripts (red) and all predicted transcripts (blue) for each *Hc* strain were plotted.
(EPS)

S2 Fig Assembled transcripts improve intron and transcript end boundaries. Paired-end read coverage (log_2_) of G217B yeast (red) and hyphae (green) mRNA is plotted for the genomic regions encompassing two (A and B) *Hc* transcripts shown. Predicted (grey) and assembled (purple) transcript structures are given above the read coverage plots with the predicted CDS regions highlighted in dark grey for predicted transcripts and dark purple for assembled transcripts. For clarity, only the strand and genomic region encompassing each transcript are displayed.
(EPS)

S3 Fig Novel transcripts exhibit a bias towards small CDS regions. Transcript length versus size of CDS was plotted for transcripts in the novel orthogroup set (n = 423; red) as well as for each transcript in the total orthogroup set (n = 6, 791; blue) per *Hc* strain. Contours are plotted based on Gaussian kernel density estimates.
(EPS)

S4 Fig Novel transcripts are expressed at low levels. For each *Hc* strain, mRNA abundances (log_2_ FPKM values) were plotted for novel transcripts (n = 423; Novels) as well as for all transcripts in the 4 strain orthogroup set (n = 6, 791; All) in yeast cells (red) and in hyphae (blue). Boxes, IQR. Whiskers, 1.5*IQR.
(EPS)

S5 Fig *Histoplasma* exhibits evolutionarily conserved yeast and hyphal transcript expression patterns. (A) Differential yeast versus hyphae (Y/H) transcript expression patterns for the 6, 791 orthogroup set for each *Hc* strain were subjected to hierarchical clustering using an uncentered Pearson’s correlation. Data are displayed in a heatmap as log_2_ differential FPKM values as indicated by the colorbar. Yellow; upregulated in yeast. Blue; upregulated in hyphae. Black; neutral in expression. This heatmap is given as S14 and S15 Data. (B–C) The number of yeast-enriched (FPKM Y/H log_2_ ≥ 1.5) (B) and hyphal-enriched (FPKM Y/H log_2_ ≤ -1.5) (C) transcripts determined for each *Hc* strain comparison are indicated. 139 yeast-enriched and 291 hyphal-enriched transcripts were identified as conserved in expression pattern across all 4 *Hc* strains and are referred to as core yeast and core hyphal transcript sets. Transcript identities of the core yeast and hyphal sets are given in S13 Data.
(EPS)

S6 Fig Yeast cells express many transcripts encoding small, putative secreted proteins. G217B, G186AR, H88, or H143 yeast mRNA abundance (log_2_ FPKM) is plotted against ORF length (number of amino acids, AA) for the core, conserved yeast-enriched 4 strain transcript set. *CBP1*, *SOD3*, *ENG1*, *GH17*/*CFP4* transcripts that have been experimentally determined to be secreted are highlighted in red and transcripts that encode ORFs predicted to be secreted are highlighted in teal. The remainder of the YPS core transcripts are shaded yellow, and transcripts that encode ORFs ≤ 200 AAs are located in a grey-shaded rectangle. For clarity ORFs > 1000 AAs (G217B n = 3; G186AR n = 2; H88 n = 3; H143 n = 1) are not shown in this figure.
(EPS)

S7 Fig Knottins have undergone an expansion in Onygenales fungi. A phylogenetic tree illustrating the conservation of knottin proteins in fungal species is shown. Species in the Onygenales order of Ascomycetes are highlighted in red (for *Hc* strains) or purple (all other Onygenales species) and the previously characterized knottin, Avr9, from *C*. *fulvum* is highlighted in blue. *Ac*; *Aspergillus clavatus*. *Af*; *Aspergillus fischeri*. *Aflavus*; *Aspergillus flavus*. *Anidulans; Aspergillus nidulans*. *An*; *Aspergillus niger*. *Ao; Aspergillus oryzae*. *At; Aspergillus terreus*. *Bd*; *Blastomyces dermatitidis*. *Cf; Cladosporium fulvum*. *Fs; Fusarium solani*. *Hc; Histoplasma capsulatum*. *Mc; Microsporum canis*. *Mg; Microsporum gypseum*. *M*. *lini*; *Melampsora lini*. *Mp; Melampsora laricis-populina*. *Pb*; *Paracoccidioides brasiliensis*. *Pc*; *Penicillium chrysogenum*. *Pl*; *Paracoccidioides lutzii*. *Pp*; *Pichia pastoris*. *Te; Trichophyton equinum*. *Tt; Trichophyton tonsurans*. *Ur; Uncinocarpus reesii*. *Hc* transcript names are truncated from ucsf_hc.01_1.X, where X = transcript name shown.
(EPS)

S8 Fig *Histoplasma* transcripts encoding knottin family members are yeast-phase enriched. Gaussian kernel density estimates of the average mRNA Y/H ratio (log_2_ FPKM) for all conserved *Hc* orthogroup transcripts (n = 6, 791) and *Hc* transcripts encoding knottin domains (n = 30) are plotted in grey or blue, respectively.
(EPS)

S9 Fig A subset of transcripts exhibit differential sizes of transcript leader regions in yeast and hyphae. (A–D) The size of the leader region (defined as the distance from the 5’ transcript end to the start of the CDS) was determined for each transcript in yeast and hyphal cells utilizing per-state transcript assemblies for each of the 4 *Hc* strains. Contours of Gaussian kernel density estimates of hyphal (Y-axes) versus yeast (X-axes) leader sizes per transcript are plotted for (A) G217B, (B) G186AR, (C) H88, and (D) H143 transcripts. A colorbar indicating the relative densities of each color on the contour plot is shown.
(EPS)

S10 Fig Transcript leader regions are more commonly extended in yeast cells. (A–D) The size of the leader region (defined as the distance from the 5’ transcript end to the start of the CDS) was determined for each transcript in yeast and hyphae utilizing per-state yeast and hyphal transcript assemblies determined for each of the 4 *Hc* strains. The difference in size of yeast and hyphal leaders was calculated per transcript (yeast leader length–hyphal leader length) and plotted based on Gaussian kernel density estimates for (A) G217B, (B) G186AR, (C) H88, and (D) H143 transcripts. Thus, transcripts that are longer in yeast cells are positive in value and transcripts that are longer in hyphal cells are negative in value. Axes are the same for all four graphs. The number of yeast and hyphal longer leaders per strain are indicated.
(EPS)

S11 Fig Northern blot analysis confirms differential transcript size between yeast and hyphal cells for a subset of longer leader transcripts. (A) Northern blots were performed to compare transcript size in G217B yeast or hyphae total cellular RNA for the yeast phase differential leader transcripts: *ucsf_hc*.*01_1*.*G217B*.*06782* and *ucsf_hc*.*01_1*.*G217B*.*04577* as well as the hyphal phase differential leader transcripts: *ucsf_hc*.*01_1*.*G217B*.*03424*, *ucsf_hc*.*01_1*.*G217B*.*11598* (*RYP3*), and *ucsf_hc*.*01_1*.*G217B*.*12059* (*RYP2*). *ucsf_hc*.*01_1*.*G217B*.*08714* (*ACT1*) was used as a loading control. Larger transcripts are highlighted on each Northern blot by brackets (B) The corresponding transcript models are shown for each differential leader transcript probed by Northern blot. mRNA log_2_ yeast read coverage is plotted in red and log_2_ hyphal read coverage is plotted in green. The assembled transcript structures are indicated above the read coverage tracks with the predicted CDS regions highlighted in dark purple.
(TIF)

S12 Fig Measurements of ribosome footprint and mRNA counts are robust between ribosome profiling biological replicates. Linear regressions of log_10_ mRNA (A–B) and ribosome footprint counts (C–D) between biological replicates are shown. Genes with summed footprint or mRNA counts in coding regions ≥ 128 between replicates are highlighted in red and those with < 128 summed counts between biological replicates are shown in black. The Pearson correlation coefficients (adjusted r values) were determined for each comparison from genes with summed footprint or mRNA counts in coding regions ≥ 128. One-way ANOVA p values were below the machine rounding threshold (< 2.2 x 10^−16^).
(TIF)

S13 Fig Comparison of translational efficiencies between *Histoplasma* yeast and hyphal cell types. (A) Log_2_ translational efficiency values of transcripts in yeast (red) and hyphae (blue) are plotted for genes where the sum between ribosome footprint and mRNA counts in coding regions ≥ 128. (B) Ribosome density values (FPKM, log_2_) for a selection of transcripts that are thought to serve basic biological functions in both cell types were plotted for yeast (red) and hyphal (blue) cells. For this figure, ribosome footprint and mRNA biological replicate data were pooled and values are pre-median normalization.
(EPS)

S14 Fig Many yeast-phase longer leader transcripts exhibit transcriptional and translational regulation. Gaussian kernel density estimates of yeast-phase TE values (A) or Y/H mRNA values (B) for G217B yeast-phase longer leader transcripts (plotted in red), hyphal-phase longer leader transcripts (plotted in green), and transcripts without differential leaders (plotted in black) are shown. (C) Yeast-phase TE values are plotted against Y/H mRNA values for G217B yeast-phase longer leader transcripts (shown in red), hyphal-phase longer leader transcripts (shown in green), and transcripts without differential leaders (shown in grey).
(EPS)

S15 Fig Categorization of yeast and hyphal longer leader transcripts with respect to their ribosome footprint density patterns using a less stringent CDS TE fold change cut-off of 1.5 fold. (A–B) Categorization of yeast (A) and hyphal (B) longer leader transcripts with respect to their ribosome footprint density patterns. Yeast and hyphal longer leader transcripts were grouped into patterns based on longer leader ribosome occupancy and CDS TE values. Categories were determined as follows: a: Ribosome density on longer leader region and decreased differential TE for CDS of longer leader transcript, b: Ribosome density on longer leader region and no change in TE for CDS of longer leader transcript, c: Ribosome density on longer leader region and increased differential TE for CDS of longer leader transcript, d: No ribosome density on longer leader region and decreased differential TE for CDS of longer leader transcript, e: No ribosome density on longer leader region and no change in TE for CDS of longer leader transcript, f: No ribosome density on longer leader region and increased differential TE for CDS of longer leader transcript. A change in CDS TE value of ≥ 1.5 fold was used to define a TE as differential in this figure.
(EPS)

S16 Fig The *MS95* promoter/leader region confers temperature responsive regulation. (A) Ribosome footprint (log_2_ RF) and corresponding mRNA (log_2_ mRNA) read coverage is shown for *MS95*. Log_2_ yeast read coverage is plotted in red and log_2_ hyphal read coverage is plotted in green. The *MS95* assembled transcript structures are indicated above the read coverage tracks with the predicted CDS regions highlighted in dark purple. The two diagonal hatch marks across the transcript structure indicate that the full transcript extends beyond what is shown in the schematic. (B) Schematic of the *MS95* promoter/leader and *GAPDH* promoter *GFP* reporter constructs. The *GAPDH* transcript is expressed and translated at similar levels in yeast and hyphae and was used as a positive control for GFP expression in 37°C and RT grown cells. *GAPDH*p, glyceraldehyde 3-phosphate dehydrogenase promoter. *CATB*t, catalase B terminator. (C) Western blot examining GFP total protein levels in cells transformed with the *MS95* –*GFP*–*CATB*t or control (*GAPDH*p–*GFP*–*CATB*t) constructs and grown at either 37°C or RT for increasing time (1 d, 2 d, 3 d). Levels of Cdc2 were probed to assess equivalency of protein loading.
(EPS)

S17 Fig *RYP2* and *RYP3* exhibit longer leader transcript forms in hyphae. Ribosome footprint (log_2_ RF) and corresponding mRNA (log_2_ mRNA) read coverage is shown for *RYP2* (A) and *RYP3* (B). Log_2_ yeast read coverage is plotted in red and log_2_ hyphal read coverage is plotted in green. The assembled transcript structures are indicated above the read coverage tracks with the predicted CDS regions highlighted in dark purple. Ryp1 and Ryp2 associate with DNA upstream of Ryp2 at 37°C in the region indicated in blue [9].
(EPS)

S1 Table A summary of paired-end RNA-seq data collected for *Histoplasma*. A sample identifier (Sample), brief sample description (Description), total number of read pairs collected (Number of Read Pairs), percentage of read pairs removed (% of Read Pairs Removed), and percentage of read pairs aligned to each genome (% of Read Pairs Aligned to Genome) for the indicated sample are given.
(XLS)

S2 Table FPKM values between biological replicate *Hc* samples are well correlated. Pearson correlation coefficients (adjusted r values) were calculated for linear regressions of log_2_ FPKM values for the orthogroup transcript set for each comparison indicated. n = 6791 for all comparisons. One-way ANOVA p values were below the machine rounding threshold (< 2.2 x 10^16^).
(XLS)

S3 Table Table of G217B longer leader transcripts that are potential developmental regulators. Longer leaders with homology to proteins with developmental roles in other fungi or transcription factor Pfam domains are listed.
(XLS)

S4 Table Table of primer name, description, and oligonucleotide sequences used in this study. (XLS)

S1 Data G217B gff3 file containing the genomic coordinates of transcript features. From the transcript assembly of combined yeast and hyphae (Y+H) paired-end sequencing data.
(GFF3)

S2 Data G186AR gff3 file containing the genomic coordinates of transcript features. From the transcript assembly of combined yeast and hyphae (Y+H) paired-end sequencing data.
(GFF3)

S3 Data H88 gff3 file containing the genomic coordinates of transcript features. From the transcript assembly of combined yeast and hyphae (Y+H) paired-end sequencing data.
(GFF3)

S4 Data H143 gff3 file containing the genomic coordinates of transcript features. From the transcript assembly of combined yeast and hyphae (Y+H) paired-end sequencing data.
(GFF3)

S5 Data G217B fasta format file of transcript sequences (Y + H). (FASTA)

S6 Data G186AR fasta format file of transcript sequences (Y + H). (FASTA)

S7 Data H88 fasta format file of transcript sequences (Y+H). (FASTA)

S8 Data H143 fasta format file of transcript sequences (Y+H). (FASTA)

S9 Data G217B fasta format file of protein sequences (Y+H). (FASTA)

S10 Data G186AR fasta format file of protein sequences (Y+H). (FASTA)

S11 Data H88 fasta format file of protein sequences (Y+H). (FASTA)

S12 Data H143 fasta format file of protein sequences (Y+H). (FASTA)

S13 Data Tab-delimited text file containing transcription expression ratios and annotations for pooled transcriptome assemblies from all four *Hc* strains. Each row corresponds to an assembled transcript orthogroup, containing at most one transcript from each of the four strains. The columns are as follows: G217B gene name: Short gene name from Inglis et al. [12] for the predicted gene(s) with additional annotations added in this work, pipe delimited. G217B Annotation: Gene annotation from Inglis et al. [12] for the predicted gene(s) with additional annotations added in this work, pipe delimited. [G217B, G186AR, H88, H143] transcript: (4 columns) assembled transcript name, corresponding to S1–S4 Data files. [G217B, G186AR, H88, H143] predicted gene: (4 columns) GSC and BROAD predicted genes overlapping the assembled transcript. Where multiple predicted genes overlap a single transcript, they are pipe (|) delimited. [G217B, G186AR, H88, H143] Pfam accession: (4 columns) Accessions for all unique Pfam-A hits to the protein sequence translated from the assembled transcript. Pipe delimited, no e-value threshold. [G217B, G186AR, H88, H143] Pfam name: (4 columns) Short domain names, corresponding to the above accessions, pipe delimited. [G217B, G186AR, H88, H143] Pfam description: (4 columns) Domain descriptions, corresponding to the above accessions, pipe delimited. [G217B, G186AR, H88, H143] signal peptide: (4 columns) "True" if Phobius predicts a signal peptide for the protein sequence translated from the assembled transcript, "False" otherwise. Enrichment: "YPS" for core yeast-enriched transcripts, "HPS" for core hyphal-enriched transcripts. Longer Leader: "Yeast" for transcripts with a longer leader in yeast for at least 3 of 4 strains, "Hyphae" for transcripts with a longer leader in hyphae for at least 3 of 4 strains. [G217B, G186AR, H88, H143] Y/H: (4 columns) median normalized log_2_ ratio of mean yeast and hyphal FPKMs as determined by eXpress for the assembled transcript.
(TDT)

S14 Data Hc orthogroup heatmap CDT file from S5 Fig. Rows are complete (4 strain) orthogroups from S13 Data and are given in clustered order. Columns are identical to S13 Data, with the addition of UNIQID, which assigns a unique integer to each orthogroup, and GID, which indicates the corresponding leaf node in the clustered dendrogram (S15 Data).
(CDT)

S15 Data Hc orthogroup heatmap GTR file from S5 Fig. For use with S14 Data in Java Treeview (if saved as "S14_Data.gtr", Java Treeview will automatically associate the CDT and GTR files).
(GTR)

S16 Data Knottin phylogenetic tree in PHB file format for S7 Fig. This file can be opened with most phylogenetic tree view programs.
(PHB)

S17 Data Protein alignment for the knottin phylogenetic tree shown in S7 Fig. in fasta file format. (FASTA)

S18 Data Genome sources for the fungal species used to build the knottin tree shown in S7 Fig. This is a tab-delimited text file with the following columns: genus, species, strain, downloaded (date downloaded), source (web address of the source of genome used).
(TDT)

S19 Data G217B gff3 file containing the genomic coordinates of transcript features. From the transcript assembly of G217B yeast (Y) paired-end sequencing data.
(GFF3)

S20 Data G217B gff3 file containing the genomic coordinates of transcript features. From the transcript assembly of G217B hyphae (H) paired-end sequencing data.
(GFF3)

S21 Data G186AR gff3 file containing the genomic coordinates of transcript features. From the transcript assembly of G186AR yeast (Y) paired-end sequencing data.
(GFF3)

S22 Data G186AR gff3 file containing the genomic coordinates of transcript features. From the transcript assembly of G186AR hyphae (H) paired-end sequencing data.
(GFF3)

S23 Data H88 gff3 file containing the genomic coordinates of transcript features. From the transcript assembly of H88 yeast (Y) paired-end sequencing data.
(GFF3)

S24 Data H88 gff3 file containing the genomic coordinates of transcript features. From the transcript assembly of H88 hyphae (H) paired-end sequencing data.
(GFF3)

S25 Data H143 gff3 file containing the genomic coordinates of transcript features. From the transcript assembly of H143 yeast (Y) paired-end sequencing data.
(GFF3)

S26 Data H143 gff3 file containing the genomic coordinates of transcript features. From the transcript assembly of H143 hyphae (H) paired-end sequencing data.
(GFF3)

S27 Data Tab-delimited text file giving mRNA and footprint FPKM values and translational efficiencies (TE) for G217B transcripts measured by ribosome profiling. Each row corresponds to an assembled transcript. The 7 annotation columns are identical to the columns of the same name in S13 Data: "G217B gene name", "G217B Annotation", "G217B transcript", "G217B predicted gene", "G217B Pfam accession", "G217B name", "G217B description", and "Longer Leader". The remaining columns are: [Y, H] [footprint, mRNA] FPKM: FPKM values for the above counts, as described in the corresponding methods. [Y, H] [footprint, mRNA] counts: Combined counts for yeast or hyphae footprint or mRNA samples passing the criteria described in "Mapping and quantification of ribosome footprint and corresponding mRNA read" methods section. Leader Category: Longer leader classification (A-F), as diagrammed in Fig 8 and the material and methods. [Y, H] TE: Median normalized log2 (footprint/mRNA) ratios of yeast or hyphae FPKMs. mRNA Y/H: Median normalized log2 (yeast/hyphae) ratio of mRNA FPKMs.
(TDT)

S28 Data TE heatmap CDT file from Fig 6D. Rows are taken from S27 Data and are given in clustered order. Columns are identical to S27 Data, with the addition of UNIQID, which assigns a unique integer to each transcript, and GID, which indicates the corresponding leaf node in the clustered dendrogram (S29 Data).
(CDT)

S29 Data TE heatmap GTR file from Fig 6D. For use with S28 Data in Java Treeview (if saved as "S28_Data.gtr", Java Treeview will automatically associate the CDT and GTR files).
(GTR)

S30 Data TE heatmap CDT file from Fig 7A. Rows are taken from S27 Data and are given in clustered order. Columns are identical to S27 Data, with the addition of UNIQID, which assigns a unique integer to each transcript, and GID, which indicates the corresponding leaf node in the clustered dendrogram (S31 Data).
(CDT)

S31 Data TE heatmap GTR file from Fig 7A. For use with S30 Data in Java Treeview (if saved as "S30_Data.gtr", Java Treeview will automatically associate the CDT and GTR files).
(GTR)

S32 Data TE heatmap CDT file from Fig 7B. Rows are taken from S27 Data and are given in clustered order. Columns are identical to S27 Data, with the addition of UNIQID, which assigns a unique integer to each transcript, and GID, which indicates the corresponding leaf node in the clustered dendrogram (S33 Data).
(CDT)

S33 Data TE heatmap GTR file from Fig 7B. For use with S30 Data in Java Treeview (if saved as "S32_Data.gtr", Java Treeview will automatically associate the CDT and GTR files).
(GTR)
