# Correction: Genome-Wide Reprogramming of Transcript Architecture by Temperature Specifies the Developmental States of the Human Pathogen *Histoplasma*

**DOI:** 10.1371/journal.pgen.1009509

**Published:** 2021-04-07

**Authors:** Sarah A. Gilmore, Mark Voorhies, Dana Gebhart, Anita Sil

The strain G184AR is incorrectly reported as G186AR throughout the paper. The authors have provided corrected versions of Table 1, Figs 1 and 2, and Supporting Information files S1, S3–S6, S9, and S10 Figs that use the correct strain name G184AR.

**Fig 1:**
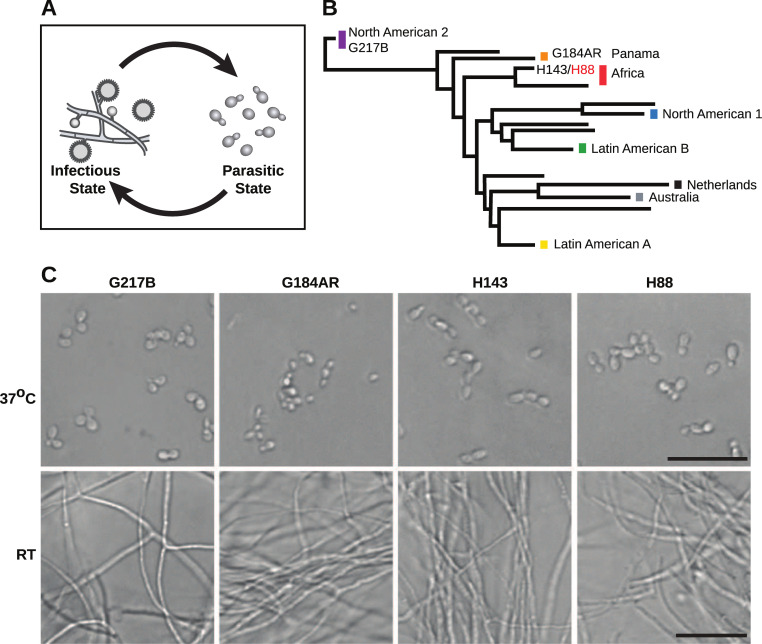
*Histoplasma* is a global human pathogen with distinct morphological states important for virulence. (A) *Histoplasma* hyphae and conidia (infectious state) respond to temperature to differentiate into budding yeast cells (parasitic state) inside of a mammalian host. (B) Phylogeny of *Histoplasma* illustrating the major lineages of this species, which are geographically and genetically distinct. This neighbor-joining tree was adapted from Katsuga et al. [7] and highlights *Histoplasma* strains selected for comparative transcriptomics (G217B, G184AR, H88, H143). H88 is highlighted in red as the *Hc* var. *duboisii* strain selected for transcriptome analysis. (C) Confocal DIC microscopy showing representative images of yeast (37°C) and hyphal (RT) cell morphologies for the 4 *Hc* strains used for comparative transcriptomics analyses. Scale bar, 20 μm.

**Fig 2:**
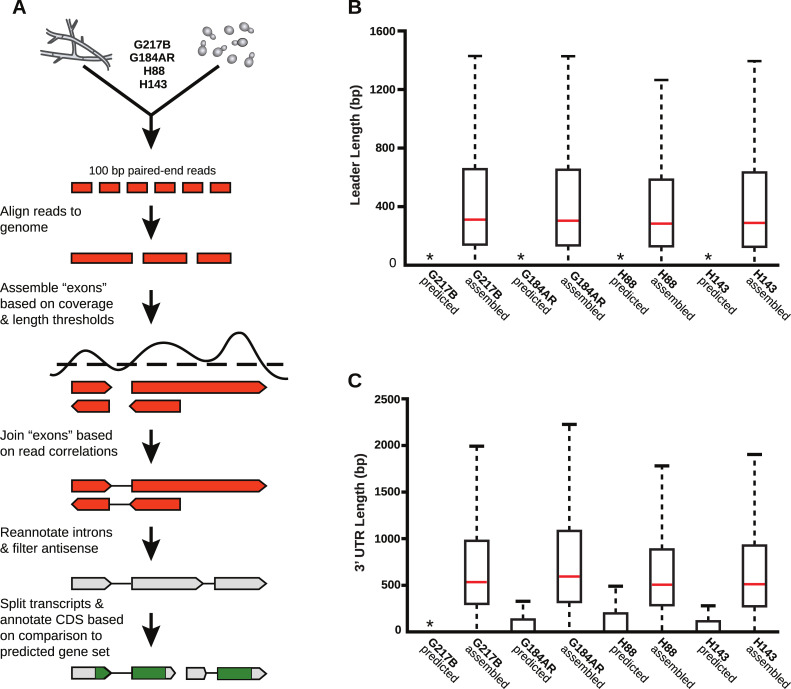
*De novo Histoplasma* transcriptome reconstruction augments transcript models. (A) Schematic of transcriptome reconstruction method. (B–C) Boxplots of the length of (B) leader regions that were defined as the distance from the 5’ transcript end to the CDS start codon or (C) 3’ UTR regions that were defined as the distance from the CDS stop codon to the 3’ transcript end were plotted for all assembled and predicted transcripts with CDS regions > 0. Boxes, interquartile range (IQR). Whiskers, 1.5*IQR. For ease of viewing, outliers are not displayed. *, indicates that the predicted transcript leader or 3’ UTR length distributions is not visible on this graph. The predicted transcript leader lengths range from 0–1652 bp and the 3’ UTR lengths range from 0–3112 bp in the 4 *Hc* strains.

**Table 1:** Comparison of numbers and orthology mapping methods of assembled and predicted transcripts.

	G217B	G184AR	H88	H143
Predicted Transcripts	11, 330	9, 233	9, 428	9, 532
Predicted Protein Coding Transcripts	11, 329	9, 229	9, 424	9, 483
Predicted InParanoid Ortholog Pairs (G217B)		7, 104	7, 122	6, 831
Predicted Mercator Orthogroups (per strain)	7, 485	7, 960	8, 610	8, 325
Assembled Transcripts	12, 313	12, 663	12, 175	12, 889
Assembled Protein Coding Transcripts	9, 580	9, 844	9, 647	9, 723
Assembled InParanoid Ortholog Pairs (G217B)		6, 708	6, 670	6, 079
Assembled Mercator Orthogroups (per strain)	7, 362	7, 659	8, 288	7, 819
1:1:1:1 Mercator Orthogroups	6, 791	6, 791	6, 791	6, 791

## Supporting information

S1 Fig *Histoplasma* assembled transcripts are longer in length than the *ab initio* predicted transcript models. The normalized kernel density estimate distributions for the length of all assembled transcripts (red) and all predicted transcripts (blue) for each *Hc* strain were plotted.
(EPS)

S3 Fig Novel transcripts exhibit a bias towards small CDS regions. Transcript length versus size of CDS was plotted for transcripts in the novel orthogroup set (n = 423; red) as well as for each transcript in the total orthogroup set (n = 6, 791; blue) per *Hc* strain. Contours are plotted based on Gaussian kernel density estimates.
(EPS)

S4 Fig Novel transcripts are expressed at low levels. For each *Hc* strain, mRNA abundances (log_2_ FPKM values) were plotted for novel transcripts (n = 423; Novels) as well as for all transcripts in the 4 strain orthogroup set (n = 6, 791; All) in yeast cells (red) and in hyphae (blue). Boxes, IQR. Whiskers, 1.5*IQR.
(EPS)

S5 Fig *Histoplasma* exhibits evolutionarily conserved yeast and hyphal transcript expression patterns. (A) Differential yeast versus hyphae (Y/H) transcript expression patterns for the 6, 791 orthogroup set for each *Hc* strain were subjected to hierarchical clustering using an uncentered Pearson’s correlation. Data are displayed in a heatmap as log_2_ differential FPKM values as indicated by the colorbar. Yellow; upregulated in yeast. Blue; upregulated in hyphae. Black; neutral in expression. This heatmap is given as S14 and S15 Data. (B–C) The number of yeast-enriched (FPKM Y/H log2 ≥ 1.5) (B) and hyphal-enriched (FPKM Y/H log_2_ ≤ -1.5) (C) transcripts determined for each *Hc* strain comparison are indicated. 139 yeast-enriched and 291 hyphal-enriched transcripts were identified as conserved in expression pattern across all 4 *Hc* strains and are referred to as core yeast and core hyphal transcript sets. Transcript identities of the core yeast and hyphal sets are given in S13 Data.
(EPS)

S6 Fig Yeast cells express many transcripts encoding small, putative secreted proteins. G217B, G184AR, H88, or H143 yeast mRNA abundance (log_2_ FPKM) is plotted against ORF length (number of amino acids, AA) for the core, conserved yeast-enriched 4 strain transcript set. *CBP1*, *SOD3*, *ENG1*, *GH17/CFP4* transcripts that have been experimentally determined to be secreted are highlighted in red and transcripts that encode ORFs predicted to be secreted are highlighted in teal. The remainder of the YPS core transcripts are shaded yellow, and transcripts that encode ORFs ≤ 200 AAs are located in a grey-shaded rectangle. For clarity ORFs > 1000 AAs (G217B n = 3; G184AR n = 2; H88 n = 3; H143 n = 1) are not shown in this figure.
(EPS)

S9 Fig A subset of transcripts exhibit differential sizes of transcript leader regions in yeast and hyphae. (A–D) The size of the leader region (defined as the distance from the 5’ transcript end to the start of the CDS) was determined for each transcript in yeast and hyphal cells utilizing per-state transcript assemblies for each of the 4 *Hc* strains. Contours of Gaussian kernel density estimates of hyphal (Y-axes) versus yeast (X-axes) leader sizes per transcript are plotted for (A) G217B, (B) G184AR, (C) H88, and (D) H143 transcripts. A colorbar indicating the relative densities of each color on the contour plot is shown.
(EPS)

S10 Fig Transcript leader regions are more commonly extended in yeast cells. (A–D) The size of the leader region (defined as the distance from the 5’ transcript end to the start of the CDS) was determined for each transcript in yeast and hyphae utilizing per-state yeast and hyphal transcript assemblies determined for each of the 4 *Hc* strains. The difference in size of yeast and hyphal leaders was calculated per transcript (yeast leader length–hyphal leader length) and plotted based on Gaussian kernel density estimates for (A) G217B, (B) G184AR, (C) H88, and (D) H143 transcripts. Thus, transcripts that are longer in yeast cells are positive in value and transcripts that are longer in hyphal cells are negative in value. Axes are the same for all four graphs. The number of yeast and hyphal longer leaders per strain are indicated.
(EPS)
