# Supplementary figures and images for: Genome-Wide Reprogramming of Transcript Architecture by Temperature Specifies the Developmental States of the Human Pathogen Histoplasma
Source: PLoS Genet. 2015 Jul 15;11(7):e1005395. doi: 10.1371/journal.pgen.1005395 (PMC4503680; doi:10.1371/journal.pgen.1005395)

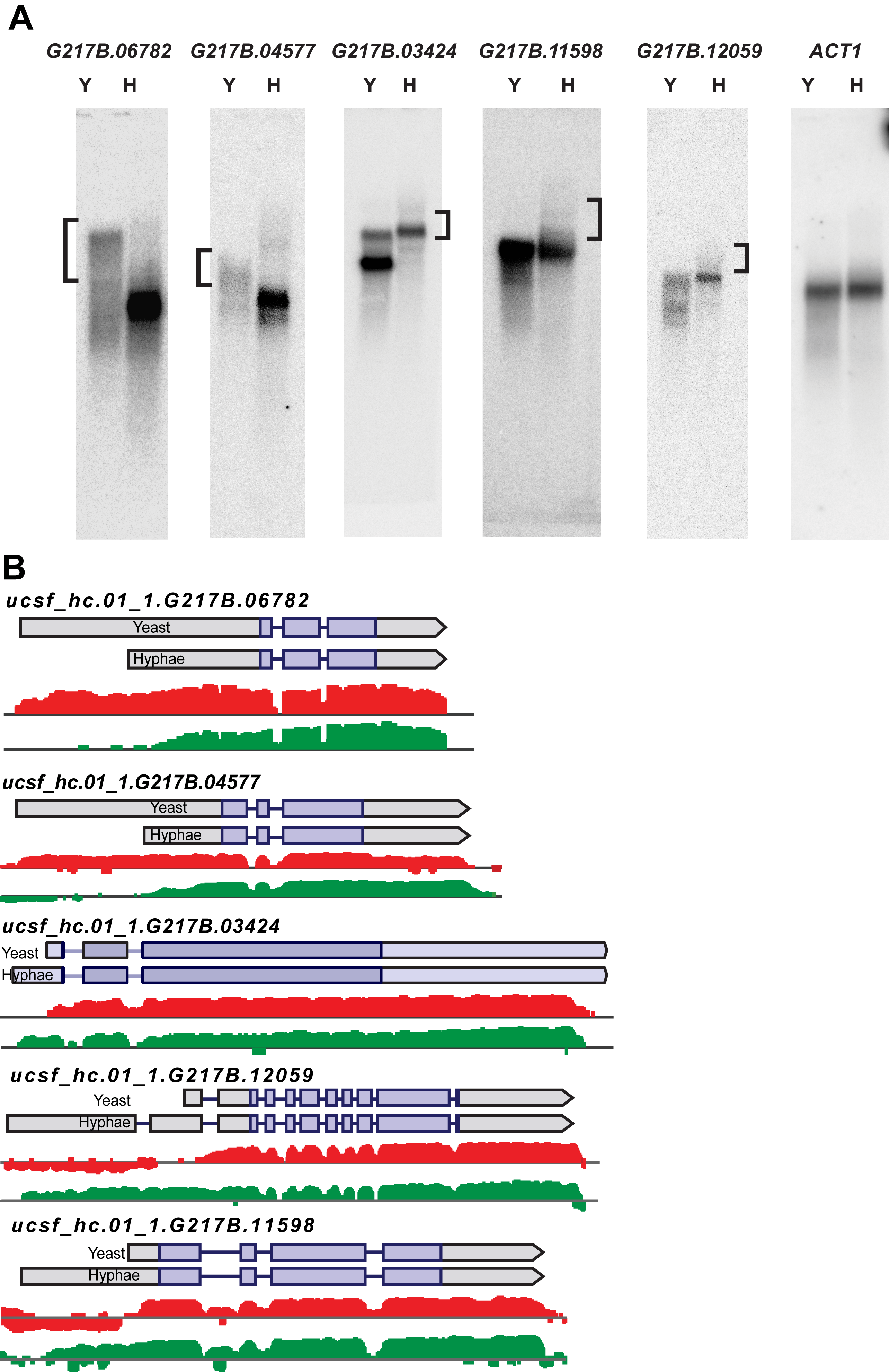

Supplement: S11 Fig — (A) Northern blots were performed to compare transcript size in G217B yeast or hyphae total cellular RNA for the yeast phase differential leader transcripts: ucsf_hc.01_1.G217B.06782 and ucsf_hc.01_1.G217B.04577 as well as the hyphal phase differential leader transcripts: ucsf_hc.01_1.G217B.03424, ucsf_hc.01_1.G217B.11598 (RYP3), and ucsf_hc.01_1.G217B.12059 (RYP2). ucsf_hc.01_1.G217B.08714 (ACT1) was used as a loading control. Larger transcripts are highlighted on each Northern blot by brackets (B) The corresponding transcript models are shown for each differential leader transcript probed by Northern blot. mRNA log2 yeast read coverage is plotted in red and log2 hyphal read coverage is plotted in green. The assembled transcript structures are indicated above the read coverage tracks with the predicted CDS regions highlighted in dark purple. (TIF) [file pgen.1005395.s011.tif]

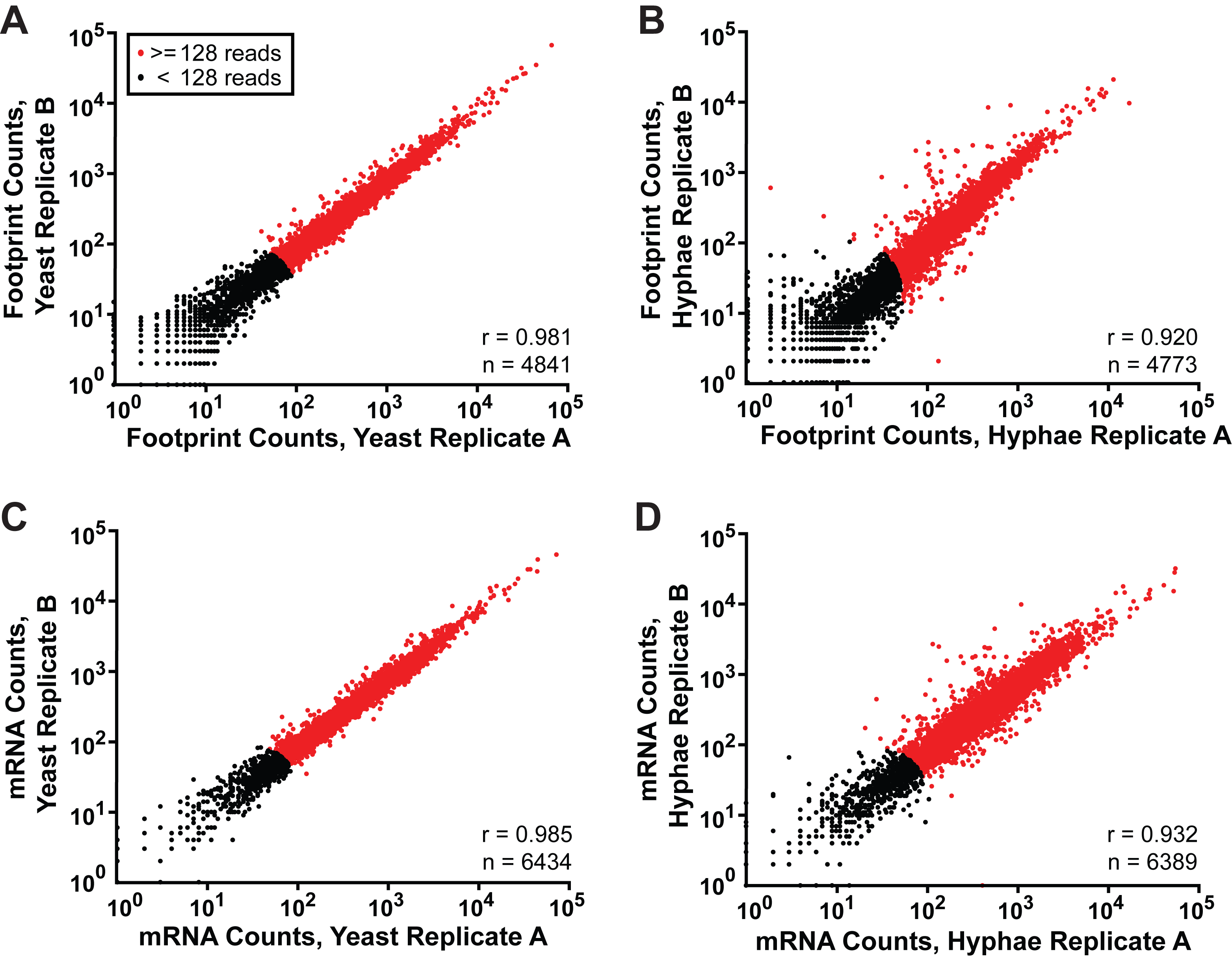

Supplement: S12 Fig — Linear regressions of log10 mRNA (A–B) and ribosome footprint counts (C–D) between biological replicates are shown. Genes with summed footprint or mRNA counts in coding regions ≥ 128 between replicates are highlighted in red and those with < 128 summed counts between biological replicates are shown in black. The Pearson correlation coefficients (adjusted r values) were determined for each comparison from genes with summed footprint or mRNA counts in coding regions ≥ 128. One-way ANOVA p values were below the machine rounding threshold (< 2.2 x 10^−16^). (TIF) [file pgen.1005395.s012.tif]
